# Patient Experience Factors and Implications for Improvement Based on the Treatment Journey of Patients with Head and Neck Cancer

**DOI:** 10.3390/cancers15082265

**Published:** 2023-04-12

**Authors:** Yoori Koo, Eunjeong Kim, Yelin Jo, Innchul Nam

**Affiliations:** 1 Department of Service Design, Graduate School of Industrial Arts, Hongik University, Seoul 04066, Republic of Korea; yrkoo@hongik.ac.kr; 2 Department of Industry-Academic Cooperation Foundation, The Catholic University of Korea, Seoul 06591, Republic of Korea; dodam.design.research@gmail.com; 3 Department of Visual Communication Design, Graduate School of Design, Hongik University, Seoul 04066, Republic of Korea; 0318ynju@naver.com; 4 Department of Otorhinolaryngology-Head and Neck Surgery, Incheon St. Mary’s Hospital, The Catholic University of Korea, Incheon 21431, Republic of Korea

**Keywords:** head and neck cancer, patient treatment journey, patient journey map, patient-centered care, barriers to patient experience, aspects of service experience, user’s integrative perspectives

## Abstract

**Simple Summary:**

Understanding the patients’ problems is essential to improve their quality of life. Through interviews and observations with patients, caregivers, and medical staff, we aimed to identify the various issues experienced by head and neck cancer patients during the treatment process and suggest improvement directions. As a result, the patients wanted to obtain comprehensive and reliable information during the treatment process. The patient also wished the medical staff could explain things easily so they could understand. For continuing long-term treatment, it was important for medical staff to encourage the patients and build a relationship with them. To successfully treat head and neck cancer patients, we need to consider what information should be delivered, how we should provide it, and how we can encourage them to support their emotions.

**Abstract:**

Based on the treatment journey, this study aimed to present insights into improving the patient-centered service experience for head and neck cancer (HNC) patients. We interviewed and observed patients, caregivers, and doctors. We conducted a qualitative content analysis and service clue analysis to identify barriers and enablers to patient care and to derive insights into the patient experience (PE). We received feedback from doctors considering the priority, importance, and feasibility of improvements and classified the insights into three service experience aspects, to suggest improvement directions. As a result, the ‘functional’ aspect of service experience stressed the importance of a comprehensive guide to the treatment process, delivery of reliable information, use of easy-to-understand terms, repeated summary explanations, the establishment of close and flexible linkages between departments, and the provision of educational programs. Regarding the ‘mechanic’ aspect, the use of large and clear visuals for patients, to easily understand the care information provided by medical staff was distinguished. In the ‘humanic’ aspect, patients’ psychological stability, trust in doctors, and doctor’s encouragement and support through maintaining a positive attitude were prioritized. This qualitative study provided integrative insights into the HNC patient experience, through the application of service design methodologies, such as a patient journey map, participatory research methods, and service experience clues.

## 1. Introduction

Patient-centeredness is one of the critical elements of high-quality care [1], and hospitals increasingly emphasize a patient-centered approach [2,3]. Patient-centered care (PCC) provides meticulous care in response to patient preferences and values, and patient opinion must be considered when making treatment decisions [1,4,5]. Understanding PE factors helps provide quality care in various respects, such as improved health outcomes, increased patient satisfaction, and reduced health costs [4,5]. PCC encompasses diverse aspects of healthcare, such as the hospital system (transitional care, coordination, care access), information and communication, and caregiver support [5]. This approach is essential to facilitate the various interactions between stakeholders, such as patients, caregivers, family members, and medical staff.

Due to the importance of PCC, many centers are trialing a user-centered approach through collaborations with designers, to improve PE [3,6]. This entails designing new processes based on a product or service for a patient-centered experience [7] and effectively identifying improvements to existing problems [3,8,9,10].

Service design aims to understand the user experience and suggest meaningful insights for a user-centered solution. It is a practical approach to PCC in healthcare [11] and includes the various stakeholders, such as the patients, caregivers, and medical staff [12]. It focuses on efficiently improving various problems in the interaction between stakeholders [7], by employing participatory research methods through patient engagement. As an effective way to engage patients in complex healthcare problem-solving processes, design ethnography, such as observation and interviews to gain understanding of the patient, can be applied to record PE and collect related data. This helps to understand the underlying meanings the patient may not express directly. A patient journey map (JM) is also a valuable method of providing medical services from a user-centered point of view, by actively managing the patient’s perspective [13,14,15,16]. Previous studies have confirmed the importance of understanding the patient journey, by translating intangible data into a concrete structure [3,8,17,18,19]. A JM is a visual representation of the sequence of a service process [11] and helps in understanding the service system and gaining insights into the journey stages [3,13,14,15,20]. A JM considers the overall process of the patient journey from a holistic viewpoint, such as actions, emotions, and touchpoints (a contact point of interaction between a user and a service provider, system, or product). It facilitates understanding and communication among stakeholders for problem-solving. A JM identifies and visualizes the patient treatment journey and analyzes the behavior, emotions, and tangible and intangible experiences through patient contact, to collect PE data using interviews or observations [3].

Meanwhile, it is crucial to understand what types of service are provided to patients to improve healthcare. Since one small and specific clue can significantly impact the evaluation of the patient’s medical service experience, service clue management is critical to improving the patient’s healthcare service satisfaction and the service experience. Service clues are related to various aspects of service experience, depending on their type. It is necessary to understand the types of clues and seek service improvement plans accordingly. Berry et al. [21] introduced service experiences, including ‘functional’, ‘mechanic’, and ‘humanic’ aspects, which contain valuable information that provide insights for creatively transforming the user service experience [22]. A ‘functional’ aspect is the technique used in providing a PE service; a ‘mechanic’ aspect is an object or environment delivered through the senses; and ‘humanic’ aspects include the interactions, attitudes, emotions, and behaviors between users [21].

Head and neck cancer (HNC) is relatively rare compared to other cancers, with limited access to treatment information. Due to the anatomical characteristics of HNC, a loss of the essential functions of eating, speaking, and breathing occurs [23]; patient quality of life deteriorates, and most experience physical and psychological atrophy [24]. HNC patients often require combinations of several treatments, such as surgery, chemotherapy, and radiation therapy. The treatment duration is long, and rehabilitation entails functional deficits after treatment, so it takes longer to return to everyday life [25]. Patients undergoing such a long treatment journey often have decreased psychological well-being and physical health. The incidence rate is high in elderly patients and people with low socioeconomic status, resulting in a poor understanding of the disease and the treatment process [26]. Therefore, providing quality, patient-centered treatment for these patients is crucial. However, few authors have conducted studies on improving the experience of HNC patients [1]. 

Thus, improving the physical and psychological well-being of patients with HNC is crucial to improving the overall patient experience, increasing satisfaction with medical services, increasing treatment adherence, and encouraging positive treatment outcomes. Therefore, we aimed to analyze the PE throughout the patient journey of patients with HNC and present a service design strategy for improving the PE at each journey stage. In this regard, the specific research questions were as follows:

RQ1. What are the enablers and barriers to patient experience at each stage of the treatment journey for patients with HNC?
RQ2. Which service design strategies be suggested to improve the experience of head and neck cancer patients at each stage of the treatment journey?

## 2. Materials and Methods

**Figure 1:**
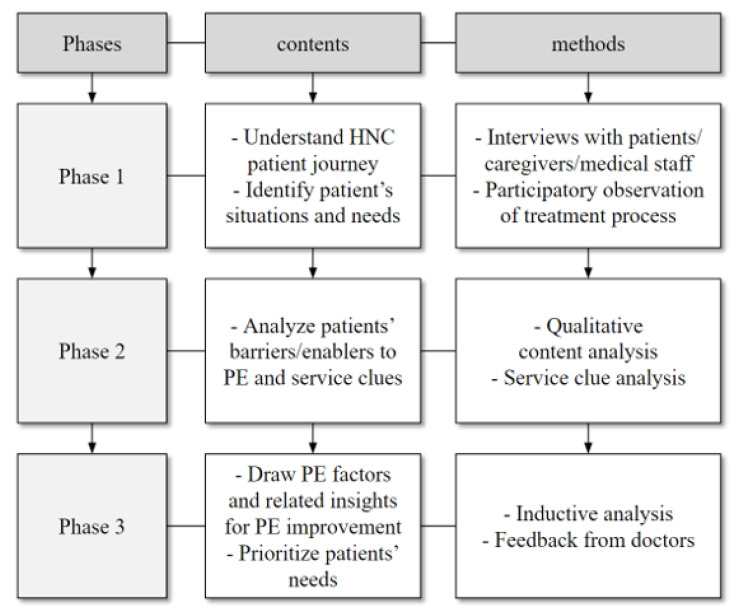
The flow chart of the research process.

Service design methods such as interviews, observations, and journey maps were used to understand the barriers and enablers (B/E) from the patient’s perspective. Through qualitative content analysis of the patient’s narratives and analysis of service clue types, the patient’s needs at each stage of the treatment journey were visually structured. Since the patient’s treatment journey includes various stakeholders, such as the patients, doctors, families, and caregivers, the opinions of doctors, caregivers, and patients were comprehensively reflected to understand the B/E of the patient’s experience. The study consisted of three phases: (1) identifying the treatment journey of HNC patients, (2) analyzing the B/Es to PE, and (3) drawing insights for improvement (Figure 1). In the first phase, we conducted nine interviews and five observations with 31 participants between August and October 2021 (Table 1). 

Interviews were conducted with doctors, patients, and caregivers, to understand the HNC treatment journey and identify specific actions, touchpoints, and emotions for each journey stage. Interviewees were directly recruited by an otolaryngology specialist from outpatients who had been diagnosed with head and neck cancer. The doctor introduced the purpose and content of the study to the patient and briefly explained the purpose and method of the interview. When the patient and guardian agreed to an interview, an in-depth interview was conducted immediately after outpatient treatment or on a different date. The interview was held in a separate private room in the hospital and lasted about an hour. The interviewees included all patients diagnosed with laryngeal, pharyngeal, or oral cancer who required surgery or were undergoing rehabilitation. The reason for this is that there is little difference in the treatment principles for each subsite, regarding the process that patients experience after treatment, problems and complications, and the needs of patients.

We also made observations during clinical counseling, to precisely understand the users’ activities, physical space, and facility layout, including the furniture, doctor –patient interactions, objects being used, and users’ attitudes and behaviors, if researcher participation was permitted. Based on the user research, a five-phased HNC treatment journey was structured. For the journey map, we identified the specific stages of the HNC treatment journey and analyzed the user’s actions, emotions, and touchpoints related to each stage. The user’s actions were indicated in the order of specific actions that occurred at each stage, and the emotions felt by the patient at each stage were described. A touchpoint is a point of contact where patients, caregivers, and medical staff interact. The touchpoints included various types, such as the pain and emotion of the patient, language to convey information, hospital system, image data, and explanation tools.

In the second phase, we took an inductive approach to analyzing the user research data. Qualitative content analysis and service clue analysis were applied to the semantic analysis of interview data, to understand the B/Es to PE. For the content analysis, following the method introduced by Graneheim and Lundman [27], we initially divided the full text of the transcript into meaning units matching the topic and purpose we wanted to know about and arranged each unit into a single sentence. A condensed meaning unit was then made by summarizing the keywords, including the manifest meaning unit, which summarizes the direct meaning, and the latent meaning unit, which grasps and interprets the latent meaning. The condensed meaning units were coded based on the core keywords of the semantic structure that was revealed directly or indirectly. The final coded units were plotted onto the JM by classifying negative and positive factors as barriers and enablers to PE. Each coding unit stage was decided considering the comprehensive relationship between the patient’s treatment process, actions, touchpoints, and emotional state. The units were analyzed to determine which service clues corresponded to understanding the characteristics of the service areas to be improved. Finally, based on the identified B/E, we derived insights to improve the PE in phase three. The patient’s B/E and interpreted insights were classified into three service experience aspects, to suggest improvement directions. We received doctor feedback to evaluate the improvement’s priority, importance, and feasibility. For the feedback, we conducted three focus group meetings (one offline and two zoom meetings) and received frequent email feedback. In the first meeting, we reviewed the insights’ appropriateness and discussed whether the patient’s core needs were reflected at each journey stage. In the second meeting, key insights were selected by comprehensively reflecting on the medical service system at the hospital site, the frequency and importance of patient needs based on the patient treatment experience, and the perspectives of medical staff. At the third meeting, the appropriateness of the selected insights and the possibility of additional selections were reviewed, and finally the insights for improvement were confirmed. Confirmed insights were highlighted with **. In the middle of the three meetings, whenever necessary, an email was sent to the medical staff to ask for opinions on the accuracy of terminology, the appropriateness of the content, and the logic of the analysis process. Feedback was reflected in the analysis results as soon as the feedback was received.

## 3. Results

### 3.1. Phase 1—Patient Journey

Interviews were conducted with one specialist and two residents, to understand the treatment process. Based on the interviews, a JM of HNC patients was created, which consisted of five stages: ‘studies for cancer diagnosis and staging’; ‘pre-operative counseling’; ‘getting operation consent’; ‘surgery and recovery’; and ‘rehabilitation and follow-up’ (Figure 1). We also conducted six interviews, five recordings, and two participatory observations with patients and caregivers, to understand the primary touchpoints, actions, and emotions during the patient’s treatment journey. 

Table 2 summarizes the patient’s primary actions, emotions, and touchpoints at each stage. Stage 1 (studies for cancer diagnosis and staging) involved four actions: recognizing symptoms, visiting the hospital/consulting, running studies for staging, and checking results/being diagnosed with cancer. As main touchpoints for this stage, the website for navigating cancer information, verbal language between patient and doctor, biopsy and imaging studies for cancer diagnosis, doctor’s attitude toward patients, and patient’s physical pain were identified. Stage 2 (pre-operative counseling) involved four actions (determining treatment methods, explaining and discussing the treatment, and deciding/planning the schedule) and seven touchpoints (evaluation report, body language used by the patient or verbal language between patient and doctor, visual material being used to explain the treatment, doctor’s attitude toward patients, patient’s pain, and hospital system to manage treatment schedule). Stage 3 (getting operation consent) involved four actions (entering the consulting room to sign the operation consent, explaining the surgical process and method, asking questions and answers regarding the consent, and signing the consent), and six touchpoints (consulting room, hand drawings, and image data used to explain the operation consent, body language and verbal language between patient and doctor, and consent paper form). Stage 4 (surgery and recovery) involved three actions (being hospitalized for surgery, recovering after surgery, and consulting other departments), and six touchpoints (doctor’s verbal language, treatment cost, visual material to check the patient’s condition, patient’s pain after the surgery, doctor’s attitude toward patients, and hospital system to support treatment cost and administrative guidance). Stage 5 (rehabilitation and follow-up) involved two actions (undergoing rehabilitation, visiting the hospital for follow-up check) and seven touchpoints (patient’s pain, an education program for patient’s self-care after discharge, hospital system for rehabilitation treatment and education, hospital location/facility, website for continuous information sharing, verbal language, and doctor’s attitude toward patients). 

The patient’s emotions showed substantial negative aspects of anxiety and worry throughout the journey. In stage 2, it was found that the patient’s trust in the doctor regarding the surgery revealed a positive desire for treatment. In stages 4 and 5, if the surgical results were good, the will to live was strongly formed as a positive emotion. 

### 3.2. Phase 2—B/E to HNC PE

For each stage of the previously created JM, a content analysis of the data collected during user research was conducted to derive meaning units, which were classified into enablers as positive factors, and barriers as negative factors. Then, each factor was classified according to the corresponding touchpoint and what type of service clue corresponded to each was analyzed. Enablers correspond to a factor that is helpful or necessary to improve the patient’s medical experience. This addresses aspects that need to be further strengthened for providing satisfying HNC PE in the future. On the other hand, barriers correspond to content where users complain of discomfort or have negative evaluations during the treatment process. This refers to the aspects that need to be improved for future PE. 

Table 3(1–5) introduce the enablers and barriers identified at each stage of the five treatment journeys. For each PE factor, the enablers and barriers related to specific actions and touchpoints at each stage were distinguished. Each factor was guided by including the coded source of the interview in parentheses. The service clue corresponding to each factor was identified and marked as (F) as functional clue, (M) as mechanic clue, and (H) as humanic clue. The final insights were derived by synthesizing enablers to overcome barriers. Each insight represents a factor that should be considered for improving the PE for each touchpoint. According to the attributes of each factor, the insights were categorized into information content, information delivery tool, information delivery method, hospital system, patient’s pain, patient’s feelings, doctor’s attitude, treatment cost, education program, and hospital environment. 

For example, considering the touchpoint ‘website’ in Table 3(1), ‘obtain reliable information from another channel’ as an enabler, ‘lack of information about relatively rare cancers’, and ‘fear of obtaining misinformation’ were found as barriers to PE in stage 1. These three factors were related to ‘obtaining information content’, and insights into the ‘need for reliable information delivery on relatively rare cancer’ could be derived. As the insight related to the subject of ‘information content’ covers the functional technique of providing the PE service, it corresponds to the functional aspect among the three service clues. 

The touchpoints of ‘patient’s emotion’, ‘anxiety and worries about life after surgery’, and ‘concerns about a long treatment journey’ were found to be barriers to HNC PE. These two barriers correspond to ‘patient’s feelings’. They can be summarized as an insight into the ‘need to alleviate anxiety and worry about the long-term treatment journey and life after surgery’. This insight represents a humanic clue, because it deals with the emotional aspect of humans.

The following insights were identified at each stage of the journey during the above analysis process.

#### 3.2.1. Stage 1

In Stage 1, from the content analysis of user research data, 13 enablers (ten functional clues, and three humanic clues) and 11 barriers (six functional clues, one mechanic clue, and four humanic clues) were identified. Taken together, 11 insights (seven functional, one mechanic and three humanic clues) were elicited, two of which were identical (marked as *). Thus, the following 10 insights were finally derived, as shown in Table 3(1): ① delivery of reliable information content about relatively rare cancers (1-5c, 1-19b, 1-20a, 1-20b); ② provision of information delivery media that patients can easily access and use anywhere (1-10, 1-19a, 1-21); ③ description of the need for pre-operative examinations and guidance on the evaluation procedure (1-12a, 1-12b, 1-13a, 1-13b, 1-14b); ④ advance notice on the opportunity to choose evaluation procedure (1-12c); ⑤ * use of terms that are easy for the patient to understand (1-7a); ⑥ establishing close links between departments and staff, to minimize schedule delays (1-5b, 1-13c, 1-16, 1-17); ⑦ information on the overall treatment process, including the cause of cancer (1-8, 1-9, 1-11, 1-14c); ⑧ alleviation of the patients’ psychological shock following cancer diagnosis (1-5a); ⑨ alleviation of anxiety and worry about the long-term treatment journey and life after surgery (1-6, 1-14a); ⑩ inducement of psychological stability and formation of a bond with the patient through information sharing and a positive attitude toward patients (1-1, 1-2, 1-4, 1-15a).

Among these, ① and ③–⑦ correspond to the ‘functional’ aspects of improving PE, such as the content and method of information delivery and the hospital system. Insight ② is related to the ‘mechanic’ aspect of improving visible elements, and ⑧–⑩ involves patient emotion and the interaction between the doctor and the patient, corresponding to the ‘humanic’ aspect.

⑤ *“The doctor didn’t use many technical terms in English but explained it in Korean, making it easier to understand and better. It was also good that the doctor didn’t just pass over something like inflammation and checked it properly.” *(1-7a)

② *“At first, I searched for cancer-related information on the Internet, but there was no detail, just bragging about the hospital they work at. There is no information at all on the internet.” *(1-10)

⑧ *“It’s the first time I’ve heard of cancer, so I wondered if it couldn’t be done without an incision, and the names of cancers were so diverse and difficult that it was hard to understand. I didn’t know anything, so I pretended to understand and said, ‘Yes, please do well’ when the professor said a lot.” *(1-1)

#### 3.2.2. Stage 2

In Stage 2, 22 enablers to strengthen PE (12 functional clues, four mechanic clues, and six humanic clues) and 12 barriers to be improved (five functional clues, three mechanic clues, and four humanic clues) were identified. The synthesized result revealed 17 insights (nine functional clues, two mechanic clues, and six humanic clues) as shown in Table 3(2): ① accurate diagnosis with clear evidence (2-50); ② comprehensive explanation and general overview of the treatment process and diagnosis with clear explanation of surgical methods, results, and rehabilitation (2-3a, 2-14, 2-16a, 2-13, 2-17b, 2-20); ③ guidance and selection of treatment methods (2-33); ④ use of easy-to-understand terms (2-35b, 2-51a, 2-51b); ⑤ provision of easy-to-understand visuals (2-17a, 2-34, 2-35a, 2-35b, 2-36, 2-38b, 2-42) and comprehensive documentation (2-17b); ⑥ services that identify and support the patient situation and concerns (2-4a, 2-4b, 2-8b, 2-21a, 2-21c, 2-24a, 2-24b); ⑦ access to successful cancer treatment cases (2-10a, 2-10b); ⑧ use of non-language communication tools (2-37a, 2-37b, 2-40); ⑨ alleviation of patient anxiety about surgery and outcomes (2-2b, 2-3a, 2-5a, 2-5b, 2-7a, 2-7b, 2-45, 2-57b, 2-57c, 2-58b); ⑩ empathy for the patient’s concerns and anxiety (2-4c, 2-6, 2-24c, 2-63); ⑪ understanding and empathy with the situation of the patient/caregiver (2-8a, 2-21b, 2-54, 2-56); ⑫ need for positive attitude and words when conveying objective facts (2-44, 2-46, 2-47a, 2-48a, 2-48c, 2-48d); ⑬ iterative explanation of information (2-18, 2-49a); ⑭ sharing cases with other patients (2-53b); ⑮ provision of text message service (2-61a, 2-61b); ⑯ trust building between doctor and patient (2-51c, 2-52, 2-55); and ⑰ emotional stability and expression of desire for long-term treatment (2-53a). 

Of these, ①–④, ⑥–⑦, and ⑬–⑮ relate to the ‘functional’ aspect regarding the content and method of information delivery and the hospital system. Points ⑤ and ⑧ relate to tangible tools corresponding to the ‘mechanic’ aspects; ⑨–⑫ and ⑯–⑰ concern the interaction between the doctor and the patient, representing the ‘humanic’ aspect.

② *“I think it would be comfortable if the doctor explained, ‘You’re in this condition now, but it will change like this in the future.’ I wonder how long the surgery will take, whether I can return to my daily life after surgery, how the evaluation test is conducted, and whether there is a ward to be hospitalized. It would be nice if the doctor could tell me about how many rounds of chemotherapy should be done after surgery.” *(2-3a)

⑤ *“It was nice that the doctor looked at my condition through an endoscope to see if I had healed well and explained it well. However, it was difficult to understand the explanation with CT images.” *(2-17a)

⑨ *“We know it’s the worst, but when the doctor speaks negatively, we lose heart. We hope to be at least a little hopeful.” *(2-2b)

#### 3.2.3. Stage 3

Stage 3 involved seven enablers (seven functional clues) and 20 barriers (five functional clues, eight mechanic clues, and seven humanic clues) regarding HNC PE improvement. Together, a total of 14 insights (seven functional clues, five mechanic clues, and two humanic clues) were derived, as shown in Table 3(3): ① securing a separated space to avoid noise and maintain privacy (3-1b); ② furniture arrangement that allows doctors and patients to interact better and focus on documents); (3-32b, 3-39b, 3-40b, 3-41b); ③ information delivery, considering the hierarchy of content (3-6b, 3-14b, 3-16b); ④ pre-information on treatment direction/postoperative pain management/intensive care in the ICU (3-7e, 3-10e, 3-23e, 3-28e); ⑤ balanced explanation by topic of information content (3-6b, 3-14b, 3-23b); ⑥ use of easy-to-understand terminology (3-13e, 3-24e); ⑦ clear explanation, pointing out the operative site (3-15e); ⑧ use of large clear visuals that are easy to understand and recognizable by older patients (3-2b, 3-30b, 3-31b, 3-32b, 3-37b, 3-41b); ⑨ alleviation of anxiety and worry (3-2b, 3-3b, 3-5b, 3-8b, 3-11b, 3-42b); ⑩ empathy and comfort from caregivers, friends, family, and medical staff (3-4b, 3-6b, 3-7b); ⑪–⑫ support of short-answer or non-verbal communication methods and tools (3-22b, 3-29b); ⑬ improvement of the content system and composition, for effective information delivery (3-16b, 3-17b); and ⑭ improved document design, so that elderly patients can easily see where to sign (3-41a). 

Of these, ③–⑦, ⑪, and ⑬ relate to the content and method of information delivery, representing the ‘functional’ aspects; ①–②, ⑧, ⑫, and ⑭ correspond to the ‘mechanic’ aspects, dealing with hospital environment, such as space and furniture arrangement; and ⑨–⑩ are related to the interaction between the doctor and the patient, representing the ‘humanic’ aspect.

③ *“The doctor gave an explanation that is difficult to understand at once, but it seemed important. But all of them were hard to understand, so I didn’t even know what was more important, and it was not even in my head. If that’s the case, I think focusing on what’s really important is better.” *(3-6b)

⑧ *“The text on the consent form was too small to read, the terms were difficult to pronounce, and the explanation took too long. I understood it at first, but later I couldn’t remember anything. I couldn’t even find a place to sign it, so the doctor marked it for me.” *(3-2b)

⑨ *“From the day I found out he had cancer, I think I am also a cancer patient. I can’t even tell you how hard it was mentally, very much.” *(3-3b)

#### 3.2.4. Stage 4

Stage 4 included 12 enablers (six functional clues, three mechanic clues, and three humanic clues) and 12 barriers (four functional clues and eight humanic clues) for HNC PE improvement. When synthesized, 14 insights (five functional, three mechanic, and six humanic clues) were drawn, and two factors were identical (marked as *). Thus, a final 13 insights were determined, as shown in Table 3(4): ① comprehensive guidance on the overall process of pre-operative treatment/postoperative progress (4-12); ② alleviation of negative feelings and psychological pains (4-4, 4-27b, 4-35); ③ communication and financial support for medical expenses (4-11, 4-26a, 4-26b, 4-27a, 4-27b, 4-28a, 4-28b, 4-29b); ④ comprehensive information guidance on the treatment progress after surgery (4-1, 4-14a, 4-14b, 4-14c, 4-31a); ⑤ use of clear visuals (4-17, 4-18); ⑥ active response to patient pain after surgery (4-7b); ⑦ preparation of measures for nutrition supply (4-5, 4-7a, 4-25a, 4-25b); ⑧ support in maintaining a positive attitude toward treatment and recovery (4-2b); ⑨ empathy and support for the caregiver’s psychological pain (4-9, 4-10); ⑩* simplified procedures through flexible link between departments (4-19, 4-20, 4-29a,4-30a, 4-30b, 4-30c, 4-31b); ⑪ encouragement and psychological support for patients after surgery (4-6); ⑫ information media that can comprehensively guide treatment procedures and content (4-13); ⑬ provision of communication media between patients/caregivers/medical staff (4-22).

Among these, ①–④ and ⑩ correspond to the content of information delivery and the hospital system, representing the ‘functional’ aspects; ⑤ and ⑫–⑬ relate to the provision and use of physical tools, engaging in the ‘mechanic’ aspect; and ②, ⑥–⑨, and ⑪,relate to patient psychology and pain and present insights for improving PE, representing the ‘humanic’ aspect.

① *“My brother was in the integrated nursing ward for a month, but I couldn’t visit even once because of COVID-19. So, the nurses in charge informed me of the patient’s condition in detail by text message every evening. I thank them so much.” *(4-12)

⑫ *“Even the slightest change in the symptoms after the surgery scared me, and I was worried about whether this was normal or a complication. I wish the doctor would let me know in advance that the symptoms will change like this in the future and what is normal.” *(4-13)

⑥ *“I don’t care about anything else, but it’s uncomfortable to breathe and talk. I need to get treated well and exercise hard from now on. My tongue is still swollen so I can’t speak fluently.” *(4-7b)

#### 3.2.5. Stage 5

Stage 5 identified 14 enablers (five functional clues, three mechanic clues, and six humanic clues) and 13 barriers (five functional clues, one mechanic clue, and seven humanic clues) for HNC PE improvement. From these, 17 insights were drawn, as shown in Table 3(5): ① encouragement to maintain a hopeful attitude toward treatment (5-3a, 5-4, 5-7, 5-8b); ② empathy and encouragement for the caregiver’s feelings (5-8a); ③ taking care of the patient’s nutritional intake and preparing countermeasures (5-1, 5-3b, 5-6b, 5-6c); ④ information related to symptom relief after surgery (5-11); ⑤ guidance on daily activity after surgery (5-22, 5-24); ⑥ flexible links and communication between departments (5-25); ⑦ providing a consultation window in case of additional inquiries (5-10); ⑧ provision of administrative assistance for guidance and reservations (5-20); ⑨ development of rehabilitation training guidelines (5-21); ⑩ provision of programs on the cause of cancer and smoking cessation/abstinence from alcohol (5-23a, 5-23b, 5-23c); ⑪ provision of a program to introduce cancer overcoming cases/medical technology trends (5-19); ⑫ convenient means of transportation to the hospital (5-16); ⑬ establishment of a comfortable and pleasant treatment environment (5-18); ⑭ development of a reliable information medium (5-17a, 5-17b); ⑮ building doctor–patient trust (5-12b, 5-13, 5-14, 5-15); ⑯ creation of an atmosphere in which patients can express their opinions comfortably (5-2); and ⑰ calm and empathetic attitude of medical staff, to relieve anxiety and tension (5-3c, 5-12a).

Of these, ④–⑪ correspond to the ‘functional’ aspects, such as information delivery content, hospital system, and educational programs; ⑫–⑭ relate to physical means, tools, and environments, representing ‘mechanic’ aspects; and ①–③ and ⑮–⑰explain the ‘humanic’ aspects, dealing with patient’s negative feelings and doctor’s empathetic attitude.

⑤ *” No detailed guidance was provided on how to behave and rehabilitate after discharge. I only heard that I should quit drinking and smoking and exercise regularly. That was all I got from the hospital.” *(5-22)

⑫ *“In fact, if it’s the same way, I’m better off closer to home.” *(5-16)

① *“The first time I visited the professor after discharge, he encouraged me to say that I had endured the difficult surgery well. He helped and encouraged me a lot. The professor did well with the surgery, so I should have hope while receiving rehabilitation treatment.” *(5-3a, 5-4, 5-7, 5-8b)

**Figure 2:**
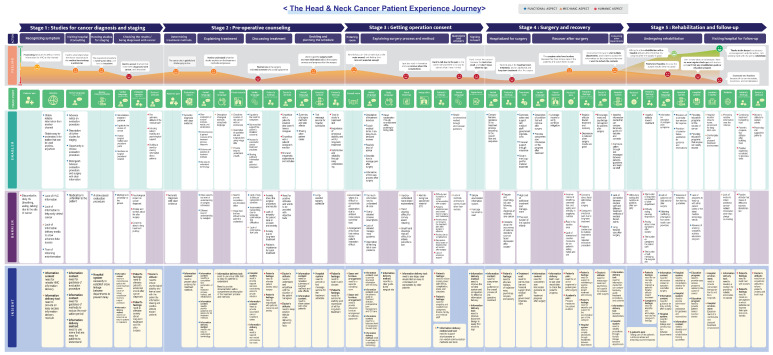
The integrated patient journey map consists of five phases.

**Figure 3:**
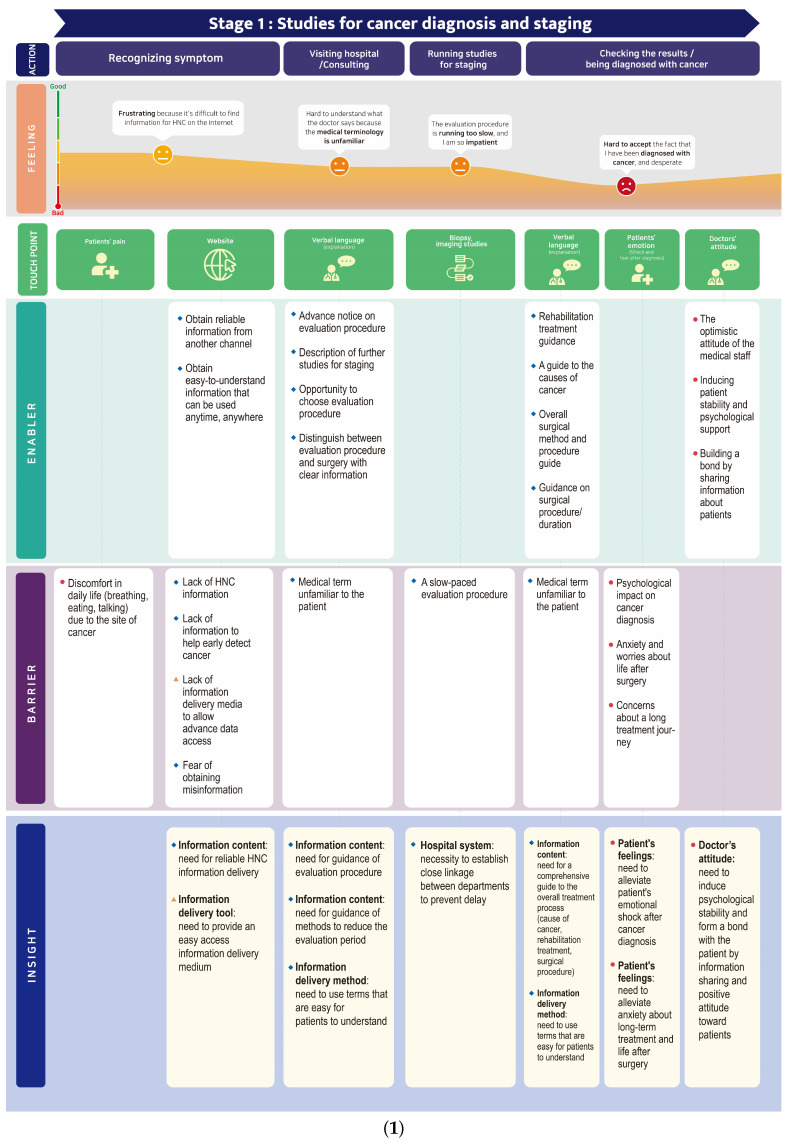

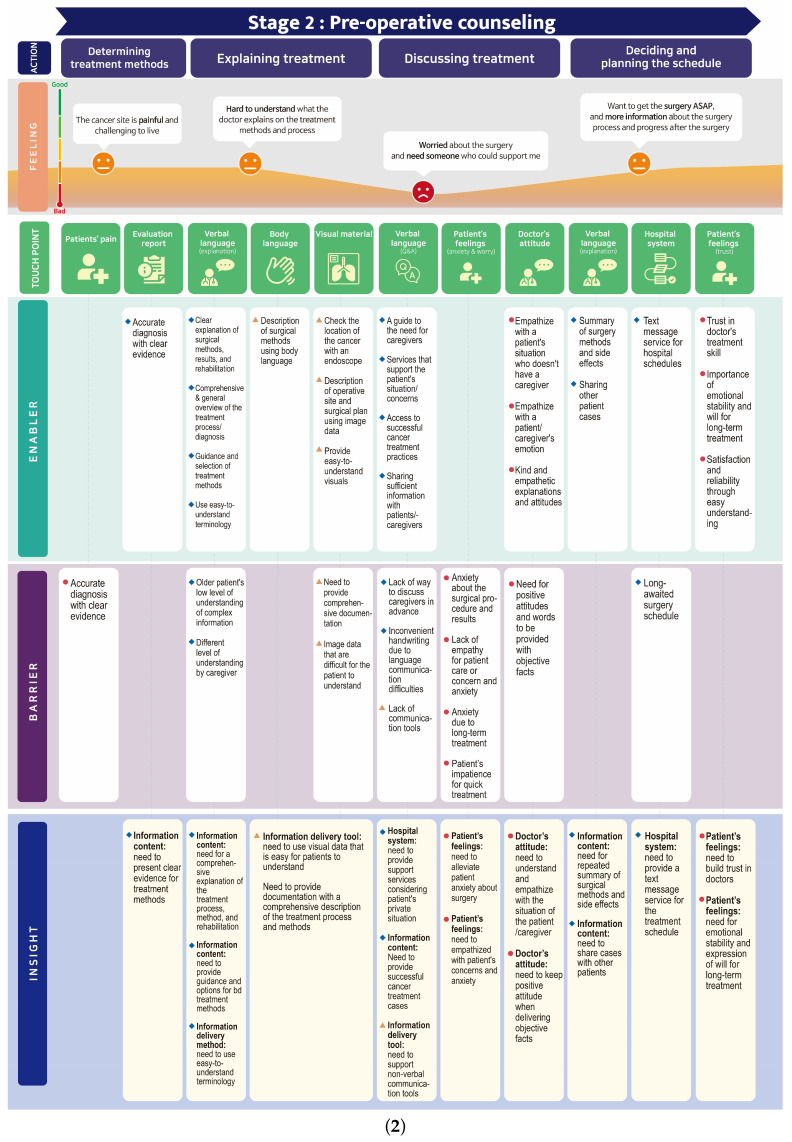

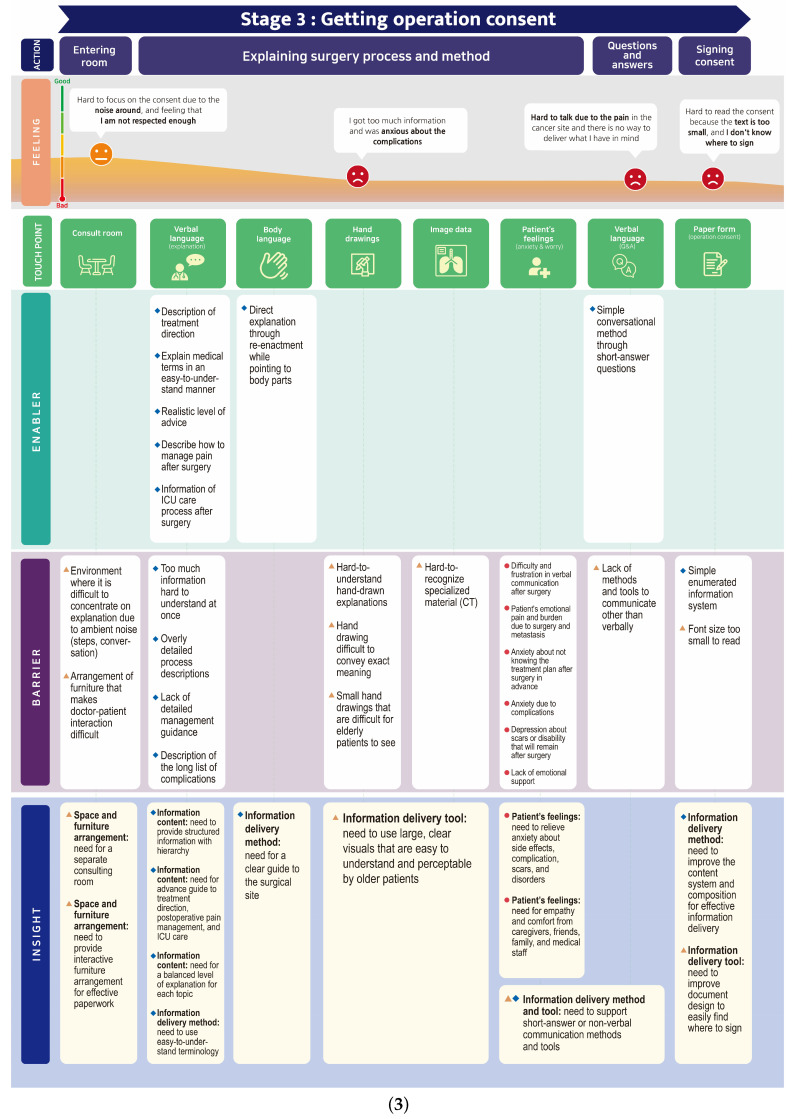

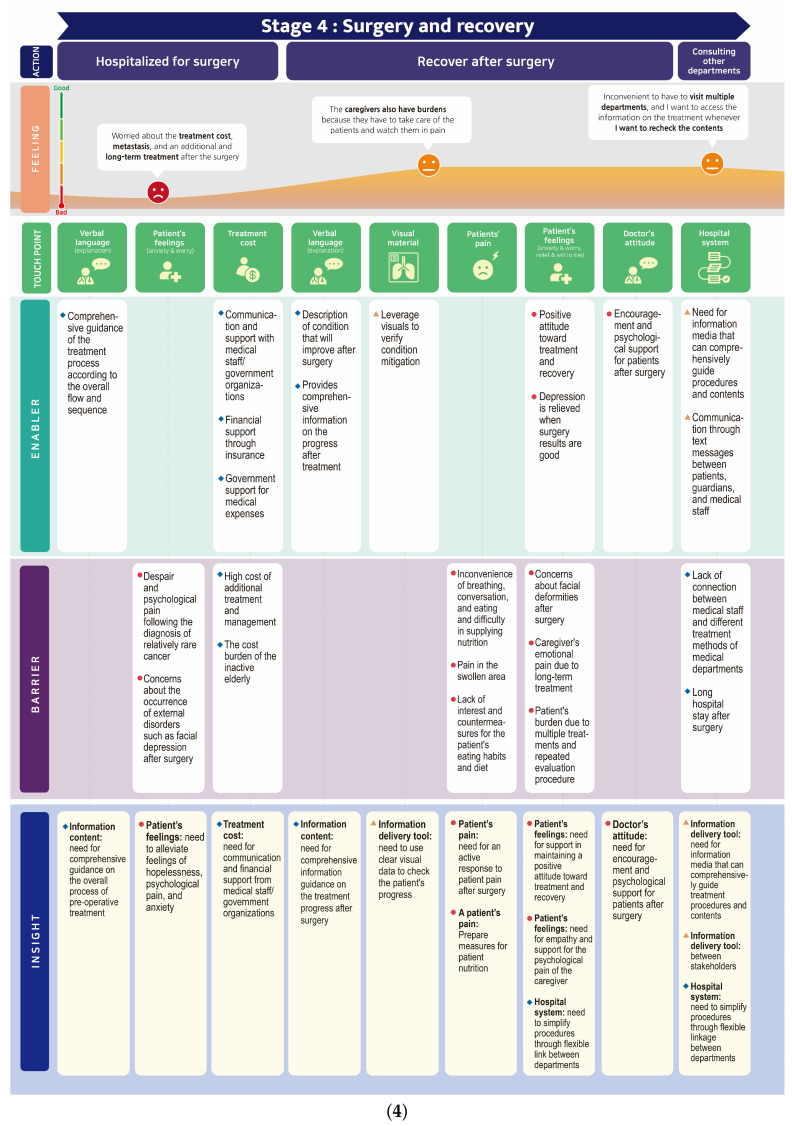

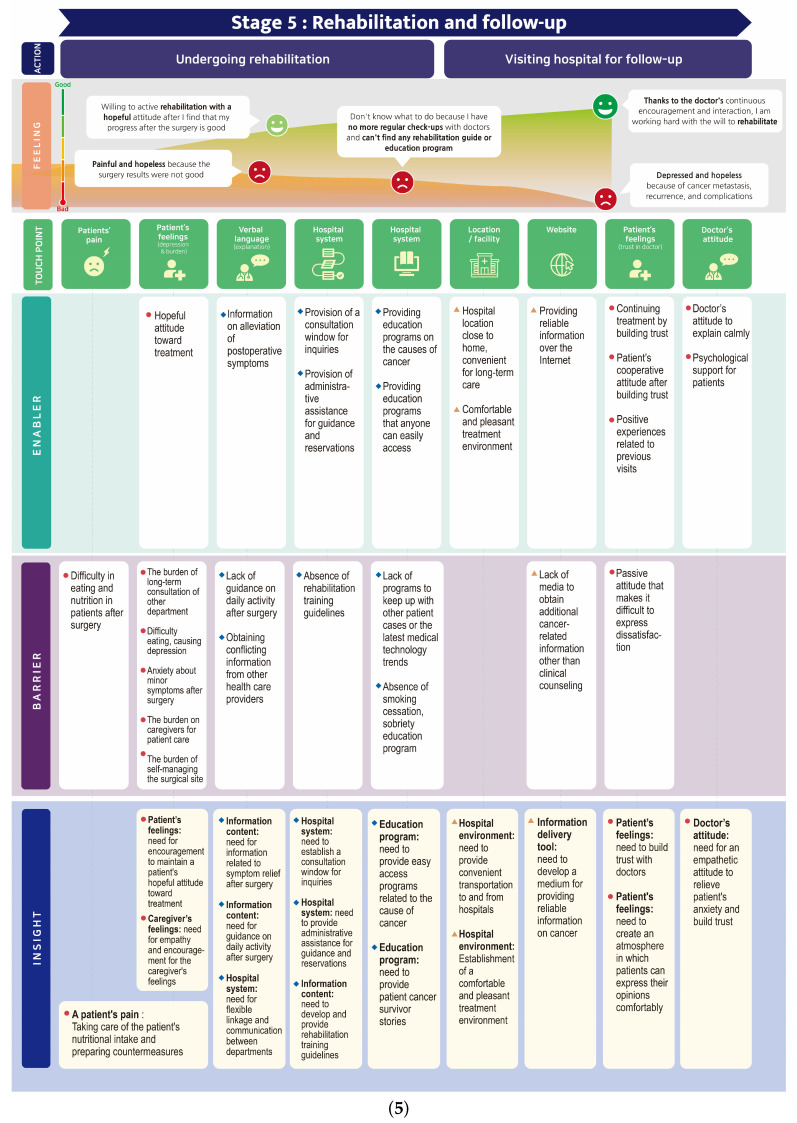
(**1**) The patient journey map: Stage 1. (**2**) Stage 2. (**3**) Stage 3. (**4**) Stage 4. (**5**) Stage 5.

The result of visually structuring using a JM, by synthesizing the above analysis, is shown in Figure 2, and the JMs for each stage correspond to Figure 3(1–5).

### 3.3. Phase 3—Insights by Service Clue Type

In this phase, by reclassifying the PE factors and the related insights identified in phase 2 into the three types of service clues, according to the attributes of the factors, we tried to identify which factors should be considered for PE improvement and what are the implications for improving HNC PE by service clue type. To this end, the ten PE factor categories classified using the attributes were divided into three service clue types. The critical insights derived at each stage of the treatment journey were summarized according to each service clue and factor type, as shown in Table 4. The implications for PE improvement from relating the PE factor derived for each of the three service clue types to the journey stage were as follows.

The ‘functional’ clue dealing with the technical aspect of providing a specific service for improving PE included five PE factors: information delivery content, information delivery method, hospital system, treatment cost, and education program. In information delivery contents, a comprehensive guidance of information related to the overall journey process consisting of five stages was required. For the information delivery method, easy-to-understand terms for patients in journey Stages 1-3, where many explanations about treatment methods and processes are made to the patient, were highlighted. In hospital system, links and communication between departments were emphasized for Stages 1, 4, and 5, to reduce patient waiting times and ensure the consistent delivery of treatment information, in consultation with other departments. In Stage 2, a customized support service for the patient that considered patient circumstances was required, along with a text message service, enabling interactions between the patient, caregiver, and medical staff and for updating the patient’s pre-operative study results. In Stage 5, an additional consultation window with the medical staff, patient continuous communication with the medical staff through administrative support, and discomfort minimization during the long-term treatment process were identified as important issues. Regarding treatment costs, government and insurance support were particularly emphasized in Stage 4. In Stage 5, educational programs were emphasized, regarding providing smoking cessation and abstinence programs, cases of overcoming cancer in other patients, and the latest HNC treatment technology trends.

The ‘mechanic’ clue, dealing with objects or environments perceptible by the senses, for improving PE was related to information delivery tools and the hospital environment. For information delivery tools, a reliable and easily accessible information delivery medium was identified as necessary in Stages 1, 4, and 5. Patients wanted as much information about treatment as possible in the early cancer diagnosis and recovery stages after surgery. In Stages 2–4, the use of clear and visible visual data that elderly patient could easily understand was emphasized. Stages 2 and 3 required a communication tool that could replace language, considering patients with difficulty in communicating verbally. In Stage 4, before and after surgery, a flexible communication tool or medium that enabled active information sharing and emotional expression among patients, caregivers, and medical staff was required. Regarding the hospital environment, in Stage 3, securing an independent counseling space for patient stability and interaction with the doctor was necessary. Thus, the arrangement of space and furniture to improve and support interactions between the patient and the doctor and help the patient focus on obtaining operation consent should be considered. In Stage 5, according to the prolonged rehabilitation treatment, it was necessary to establish convenient means of transportation and a comfortable treatment environment, considering patient convenience.

The ‘humanic’ aspect of PE dealing with user interactions, attitudes, emotions, and behaviors included needs related to the patient’s pain, patient’s feelings, and doctor attitudes. In patient’s pain, attention to nutrition supply, preparation of countermeasures, and active response to patient pain after surgery were required in Stages 4 and 5. Regarding patient’s feelings, active support and encouragement from caregivers and medical staff were needed. It was also necessary for the patient to understand the treatment journey in advance, by guiding them through detailed information for each stage, to alleviate negative emotions and maintain emotional stability. Empathy and encouragement of the caregiver’s grievances about caring for the patient after discharge were also crucial in Stages 4 and 5. In Stages 2 and 5, maintaining a positive attitude toward treatment by building trust with the doctor helps increase patient satisfaction with the PE. For doctor’s attitude, at all stages of the journey, except for Stage 3, the positive attitude of the medical staff, empathy, and encouragement of the patient played a vital role in the PE.

Above, we considered the PE factors and related insights that should be considered to improve HNC PE by service clue type. The insights for each stage of the journey presented in Table 4 reflect the perspectives of patients, caregivers, and medical staff involved in the HNC treatment journey, and it can be seen that various factors should be considered for each service clue.

We conducted three focus group meetings with HNC doctors to identify the relative importance of the factors that should be considered for improving HNC PE. We received feedback on the priorities and importance of the PE factors and related insights from two highly experienced HNC doctors, who both closely directly or indirectly interact with patients and fully understand their situation and needs. As a result, six insights were evaluated as necessary for the functional clue about information content. Three insights for information delivery methods, two for the hospital system, and two for the education program were evaluated as significant. Regarding the mechanic clue, the ‘use of large and clear visuals that are easy to understand and recognizable by older patients’ was evaluated as necessary for the information delivery tool. For the humanic aspect, three insights related to the patient’s feelings and three insights related to the doctor’s attitude were each evaluated as significant. The PE factors evaluated as relatively necessary during the feedback are marked with ** in Table 4.

Considering these feedback results, the information content that patients acquire during the treatment process is critical to improving the experience of HNC patients. In addition to the method of delivery of this information content, the painful emotions felt by patients during the treatment process, and the positive attitude of medical staff who respond appropriately to them can also be interpreted as key.

In summary, the early stages of the journey (S1–S3) involved the patient’s cancer diagnosis and treatment method delivery (Table 4). Thus, using terms that the patient could easily understand or using visual materials was necessary. Comprehensive guidance for the overall treatment process must be provided from the early stage of the journey. In the later stages (S4–S5) related to the rehabilitation after surgery, as the frequency of contact between the doctor and the patient decreases, the burden on the patient to self-manage increases. Therefore, the preparation of rehabilitation guidelines and educational programs was emphasized. Encouragement and empathy for patient anxiety were required at all journey stages. The need for an information delivery medium that the patient can always find easily was highlighted (Figure 3. (1)-(5)).

## 4. Discussion

In this study, the following results were derived through a three-phased process. In phase 1, user interviews and participatory observation identified a five-staged HNC treatment journey process. For each stage, relevant user experiences were collected, including main actions, touchpoints, and patient emotions.

In phase 2, the barriers and enablers of PE were analyzed for each journey stage through content analysis of the collected user research data. As a result, ten insights were derived from 13 enablers and 11 barriers in the first stage. In stage 2, 17 insights emerged from 22 enablers and 12 barriers. In stage 3, 14 insights were derived from seven enablers and 20 barriers. In stage 4, 13 insights were identified from 12 enablers and 12 barriers. In stage 5, 17 insights were derived from 14 enablers and 13 barriers.

In phase 3, the insights derived from each stage were classified according to the three service clue types and categories of PE factors. The ‘functional’ aspect of service experience included five categories and 40 insights, stressing the importance of a comprehensive guide to the treatment process, delivery of reliable information, use of the easy-to-understand terms, repeated summary explanation, the establishment of close and flexible linkages between departments, and provision of educational programs (smoking/alcohol, and cancer survivor patient stories). Regarding the ‘mechanic’ aspect, two categories and 15 insights were included, such as using large and clear visuals that patients can understand easily. Three categories and 23 insights were included in the ‘humanic’ aspect, highlighting patient’s psychological stability, trust in doctors, and doctor’s encouragement and support by maintaining a positive attitude.

First, for the ‘functional’ aspect, regarding information content, the overall process of the treatment journey for HNC patients should be guided in the early stages of the journey. As framing the holistic picture of the journey from the patient’s perspective (who must undergo a long-term treatment process) can help maintain emotional stability, the information content a patient needs at each journey stage is different. Thus, the information provided at each stage should be classified. An integrated database should also be established to provide the information for each category and to patients in a JM, where the treatment journey can be easily identified.

Second, as part of the ‘functional’ aspect, to maintain long-term and continuous treatment, educational programs should be provided in which patients can participate or receive information after discharge. Through providing a rehabilitation guide booklet and smoking cessation and sobriety education programs that can be referenced after discharge, information for continuous rehabilitation and self-management should be delivered to patients needing psychological support.

Third, with regard to the ‘mechanic’ aspect, stages 1-3 are when a patient first encounters HNC and is inundated with information. For elderly patients with reduced memory, a means to understand and accept information more efficiently is needed. As it is difficult for these patients to understand treatment information verbally, regarding information delivery methods and tools, large and clear visual data, such as human body models or illustration images, should be used as additional explanatory tools.

Fourth, regarding the ‘humanic’ aspect, HNC is a relatively rare cancer that requires long-term and complex treatment, and most patients experience depression and anxiety. To support patient emotions and to alleviate patient anxiety and worries, as well as to encourage a hopeful attitude throughout the treatment process, a video about overcoming cancer or a community for HNC patients could be provided for information exchange and psychological support between patients.

We conducted a convergence study of the medicine and service design by applying the JM methodology. As a result, we could reveal PE barriers and enablers that medical staff were perhaps unaware of. In addition, it was possible to derive realistic improvements through close interaction with the medical staff. As a result of our study, various items at each stage of the HNC patient’s journey were identified as factors related to patient experience improvements, including functional aspects such as information and systems, mechanic aspects related to specific objects or facilities to support function, and humanic aspects such as the emotions and attitudes of patients and caregivers. This aligns with previous research [5], which revealed that PCC includes various aspects, such as the hospital system, patient–doctor information and communication, and caregiver support. Furthermore, the significance of this study rests in it identifying and introducing more specific factors for PE improvement. In addition, using a JM, the patient interview and observation data were embodied and visually displayed for each stage of the journey. This helped in understanding the intangible patient needs data. This shows that our results are meaningful, as a case study supporting the assertion of previous studies [3,8,17,18,19] that a JM helps convert intangible data into concrete structures. It also supported the usefulness of JMs as a methodology for improving health care.

Since this study conducted interviews with a small number of patients and caregivers, it is difficult to generalize the results. However, a qualitative research analysis was employed to discover in-depth insights into the patients, by conducting repeated observations and interviews over the journey stages for a small group of users who fit the purpose of the study. As Nielsen et al. [28] mentioned, rather than conducting a single study with many people, it is more effective to repeatedly collect and evaluate data with a small number of people, thus supporting the approach of this study. Lastly, this study was targeted at HNC patients, and the research scope could be expanded to other cancer fields or general cancer patients.

## 5. Conclusions

Applying service design to the medical field is a critical strategy for providing quality care from a patient’s perspective. Since the patient’s journey is complex and includes diverse aspects, it is essential to interpret the various viewpoints and complex data delicately. A JM is a valuable tool for understanding the relationships and patterns between meanings, by visually structuring complex patient needs. Thus, it is of great help to holistically understand where and how the patient’s treatment experience occurs for each journey. Employing a qualitative methodology that collects patient needs through interviews and observations and systematically analyzing the contents through an inductive approach can provide more in-depth insights than the quantitative measurement of patient satisfaction. The results of this study can be used as reference materials for researchers interested in creative methodologies for improving PE.

By applying the creative methodology of service design, we employed three integrated perspectives to improve the experience of HNC patients. First, rather than dividing the treatment journey of HNC patients into fractured stages, the overall journey flow was examined in an integrated manner, to identify the changes and differences in the patient’s needs at each stage. Second, to improve HNC PE, the perspectives of caregivers and medical staff, and the patient’s perspectives were, directly and indirectly, reflected through a participatory method. Third, to identify the implications for improvement, three aspects of technical support, tangible tools, and human interactions were integrated and viewed, by applying three service experience clues: functional, mechanic, and humanic.

The study’s findings could be applied to real patient-centered treatment. Patients can easily understand the treatment process, maintain a good doctor–patient relationship, and actively participate in their treatment. This might effectively improve the treatment outcomes of difficult-to-treat HNC. It could be widely applied to cancers other than HNC, or other diseases. In that case, the efficiency of medical care would be increased, while maintaining the doctor–patient relationship in the entire medical field.

## Figures and Tables

**Table 1:** The user research methods and users involved.

User Research Method	No.	Users Involved (Gender, Age)	Patient’s Cancer Status Therapy	Date
In-depth Interview	1	Resident (female, 31)	-	27 August 2021
	2	Specialist (male, 45)	-	10 September 2021
	3	Resident (female, 31)	-	10 September 2021
	4	Patient (male, 65)/Caregiver (female, 39)	Oral cavity cancer (T2N0M0, stage II), surgery only	27 August 2021
	5	Patient (male, 56)/Caregiver (female, 54)/Specialist (male, 45)	Nasal cavity cancer (T4N0M0, stage IV), surgery with chemoradiation therapy	27 August 2021
	6	Patient (male, 70)/Caregiver (female, 68)	Oropharynx cancer (HPV+, T2N1M0, stage I), surgery with radiation therapy	27 August 2021
	7	Patient (male, 56)/Caregiver (female, 60)/Specialist (male, 45)	Oral cavity cancer (T2N2bN0, stage IVA), surgery with radiation therapy	1 October 2021
	8	Patient (male, 70)/Caregiver (female, 68)	Larynx cancer (T2N0M0, stage II), surgery only	7 October 2021
	9	Patient (female, 74)/Specialist (male, 45)	Oral cavity cancer (T1N0M0, stage I), surgery only	15 October 2021
Observation	1	Patient (male, 65)/Caregiver (male, 39)/Specialist (male, 45)	Larynx cancer (T3N1N0, stage III), surgery with radiation therapy	27 August 2021
	2	Patient (male, 48)/Caregiver (male, 49)/Specialist (male, 45)	Salivary gland cancer (T2N2aM0, stage IVA), surgery with chemoradiation therapy	27 August 2021
	3	Patient (male, 70)/Caregiver (female, 68)/Resident (female, 31)	Hypopharynx cancer (T3N1M0, stage III), surgery with chemoradiation therapy	10 September 2021
	4	Patient (male, 56)/Caregiver (female, 60)/Specialist (male, 45)	Nasopharynx cancer (T2N1M0, stage II), chemoradiation therapy	5 October 2021
	5	Patient (female, 74)/Specialist (male, 45)	Oropharynx cancer (HPV-, T3N1M0, stage III), surgery with radiation therapy	15 October 2021

**Table 2:** The five stages of the patient journey.

Category	Stage 1	Stage 2	Stage 3	Stage 4	Stage 5
Description	Studies for cancer diagnosis and staging	Pre-operative counseling	Getting operation consent	Surgery and recovery	Rehabilitation and follow-up
Action	(S1-1) Recognizing symptoms	(S2-1) Determining treatment methods	(S3-1) Entering room	(S4-1) Being hospitalized for surgery	(S5-1) Undergoing rehabilitation
	(S1-2) Visiting hospital/ consulting	(S2-2) Explaining the treatment	(S3-2) Explaining surgical process and method	(S4-2) Recovering after surgery	(S5-2) Visiting hospital for follow-up check
	(S1-3) Running studies for staging	(S2-3) Discussing the treatment	(S3-3) Questions and answers	(S4-3) Consulting other departments	-
	(S1-4) Checking results/ being diagnosed with cancer	(S2-4) Deciding and planning the schedule	(S3-4) Signing consent	-	
Emotion	- Shock and fear	- Anxiety - and worry - Trust	- Anxiety - and worry	- Anxiety - and worry - Relief - and will to live	- Relief - and will to live - Depression - and burden - Trust
Touch point	- Patient’s pain - Website - Verbal language - Biopsy, - imaging studies - Doctor’s attitude	- Patient’s pain - Evaluation report - Body language - Visual material - Verbal language - Doctor’s attitude - Hospital system	- Consulting room - Body language - Hand drawings - Image data - Verbal language - Paper form	- Verbal language - Treatment cost - Visual material - Patient’s pain - Doctor’s attitude - Hospital system	- Patient’s pain - Verbal language - Hospital system - Education program - Location/facility - Website - Doctor’s attitude

**Table 3:** (1) Journey Stage 1: studies for cancer diagnosis and staging. (2) Journey Stage 2: pre-operative counseling. (3) Journey Stage 3: getting operation consent. (4) Journey Stage 4: surgery and recovery. (5) Journey Stage 5: Rehabilitation and follow-up.

(1)				
**Action**	**Touchpoint**	**Enablers**	**Barriers**	**Insights**
(S1-1) Recognizing symptom	Patient’s pain	-	(H) Discomfort in daily life (breathing, eating, talking) according to the location of head and neck cancer	-
	Website	(F) Obtain reliable information from another channel (F) Obtain easy-to-understand information that can be used anytime, anywhere	(F) Lack of information about relatively rare cancers (F) Lack of prior information for early detection of cancer (M) Lack of information delivery media that allow information to be researched in advance (F) Fear of obtaining misinformation	① Information content: reliable information delivery on relatively rare cancers (F) ② Information delivery tool: provide an information delivery medium that patients can easily access and use anywhere (M)
(S1-2) Visiting hospital /consulting	Verbal language (explanation)	(F) Advance notice on the evaluation procedure (F) Description of further studies for staging (F) Opportunity to choose evaluation procedure (F) Distinguish between evaluation procedure and surgery, and provide clear information on it	(F) Medical term unfamiliar to the patient	③ Information content: guidance and explanation of the need for evaluation procedure (F) ④ Information content: guidance on methods that can shorten the evaluation period (F) ⑤ Information delivery method: use terms that are easy for patients to understand (F)
(S1-3) Running studies for staging	Biopsy, imaging studies	-	(F) A slow-paced evaluation procedure	⑥ Hospital system: establish close linkage between departments to prevent delay (F)
(S1-4) Checking the results/being diagnosed with cancer	Verbal language (explanation)	(F) Guidance on method and direction of rehabilitation treatment after surgery (F) A guide to the causes of cancer (F) Overall surgical method and procedure guide (F) Guidance on surgical procedure and duration	(F) Medical term unfamiliar to the patient	⑦ Information content: comprehensive guidance on the cause of cancer, postoperative rehabilitation treatment methods, surgical procedures and duration, and overall treatment process (F) ⑤ Information delivery method: use terms that are easy for patients to understand (F)
	Patient’s feelings (shock and fear)	-	(H) Psychological impact on cancer diagnosis (H) Anxiety and worries about life after surgery (H) Concerns about a long treatment journey	⑧ Patient’s feelings: alleviate the psychological shock of patients following a cancer diagnosis (H) ⑨ Patient’s feelings: alleviate anxiety and worry about long-term treatment journey and life after surgery (H)
	Doctor’s attitude	(H) The optimistic attitude of the medical staff (H) Inducing patient stability and psychological support (H) Building a bond by sharing information about patients	-	⑩ Doctor’s attitude: induce psychological stability and form a bond with the patient by information sharing and a positive attitude toward patients (H)
(2)				
(S2-1) Determining treatment methods	Patient’s pain	-	(H) Pain at the site of cancer	-
	Evaluation report	(F) Accurate diagnosis with clear evidence	-	① Information content: present clear evidence for treatment methods (F)
(S2-2) Explaining treatment	Verbal language (explanation)	(F) Clear explanation of surgical methods, results, and rehabilitation (F) Comprehensive explanation and general overview of the treatment process and diagnosis (F) Guidance and selection of treatment methods (F) Use easy-to-understand terminology	(F) Lack of understanding of older patients of unfamiliar and complex content (F) Different levels of understanding by caregiver	② Information content: a comprehensive explanation of the treatment process, method, and rehabilitation (F) ③ Information content: provide guidance and options for bd treatment methods (F) ④ Information delivery method: use terminology that is easy for the patient/caregiver to understand (F)
	Body language	(M) Description of surgical methods using body language	-	⑤ Information delivery tool: use visual data that is easy for patients to understand/provide documentation with a comprehensive description of the treatment process and methods (M)
	Visual material	(M) Check the location of cancer with an endoscope (M) Description of operative site and surgical plan using image data (M) Provide easy-to-understand visuals	(M) Need to provide comprehensive documentation (M) Image data that are difficult for the patient to understand	
(S2-3) Discussing treatment	Verbal Language (Q & A)	(F) A guide to the need for caregivers (F) Services that identify and support the patient’s situation and concerns (F) Access to successful cancer treatment practices (F) Sharing sufficient information with both patients and caregivers	(F) Lack of way to discuss with caregivers in advance (F) Inconvenient handwriting, due to language communication difficulties (M) Lack of communication tools	⑥ Hospital system: provide support services that consider the individual circumstances of the patient (F) ⑦ Information content: provide successful cancer treatment cases (F) ⑧ Information delivery tool: support non-language communication tools (M)
	Patient’s Feelings (anxiety & worry)	-	(H) Anxiety about the patient’s surgical procedure and results (H) Lack of empathy for patient care or concern and anxiety (H) Anxiety and worries from long-term treatment (H) Patient’s impatience for quick treatment	⑨ Patient’s feelings: alleviate patient anxiety about surgery and outcomes (H) ⑩ Patient’s feelings: empathy for the patient’s concerns and anxiety (H)
	Doctor’s attitude	(H) Attitude toward understanding and empathizing with the situation of a patient without a caregiver (H) Empathize with the situation and psychological aspects of the patient or caregiver (H) Kind and empathetic explanations and attitudes	(H) Need for positive attitudes and words to be provided with objective facts	⑪ Doctor’s attitude: understand and empathize with the situation of the patient/caregiver (H) ⑫ Doctor’s attitude: positive attitude and words when conveying objective facts (H)
(S2-4) Deciding and planning the schedule	Verbal language (explanation)	(F) Summary of surgery methods and side effects (F) Sharing other patient cases	-	⑬ Information content: repeated summary explanations of surgical methods and side effects (F) ⑭ Information content: share cases with other patients (F)
	Hospital system	(F) Text message service for hospital schedules	(F) Long-awaited surgery schedule	⑮ Hospital system: provide a text message service for the treatment schedule (F)
	Patient’s feelings (trust)	(H) Building trust in doctors’ experience (H) Importance of emotional stability and will for long-term treatment (H) Satisfaction and reliability through easy understanding		⑯ Patient’s feelings: build trust in doctors (H) ⑰ Patient’s feelings: emotional stability and expression of will for long-term treatment (H)
(3)				
(S3-1) Entering room	Consult room	-	(M) Environment where it is difficult to concentrate on explanation, due to ambient noise (steps, conversation) (M) Arrangement of furniture that makes doctor–patient interaction difficult	① Space and furniture arrangement: a separate medical space (M) ② Space and furniture arrangement: improve furniture arrangement, so that doctors and patients can face each other and focus on paperwork (M)
(S3-2) Explaining the surgery process and method	Verbal language (explanation)	(F) Description of treatment direction (F) Explain medical terms in an easy-to-understand manner (F) Realistic level of advice (F) Describe how to manage pain after surgery (F) Information on intensive care in the ICU after surgery	(F) A large amount of information that is difficult to hear and remember at once (F) Explanation of the process is too detailed and specific (F) Lack of detailed management guidance (F) Comprehensive and lengthy description of complications	③ Information content: information delivery considering the hierarchy and system of information content (F) ④ Information content: prior guidance on treatment direction, postoperative pain management, and intensive care in the ICU (F) ⑤ Information content: a balanced explanation by topic of information content (F) ⑥ Information delivery method: use easy-to-understand terminology (F)
	Body language	(F) Direct explanation through re-enactment, while pointing to body parts	-	⑦ Information delivery method: explanation pointing out the operative site (F)
	Hand drawings	-	(M) Hard-to-understand hand-drawn explanations (M) Hand drawing makes it challenging to convey the exact meaning (M) Small hand drawings that are difficult for elderly patients to see	⑧ Information delivery tool: use large, clear visuals that are easy to understand and recognizable by older patients (M)
	Image data	-	(M) Hard-to-recognize specialized material (CT)	
	Patient’s feelings (anxiety and worry)	-	(H) Difficulty and frustration in communication, due to inability to communicate verbally (H) Patient’s psychological burden for surgery (H) Patient’s psychological pain and burden due to metastasis (H) Worry and anxiety about not knowing the treatment plan after surgery in advance (H) Anxiety due to complications (H) Depression about scars or disability that will remain after surgery (H) Lack of emotional support	⑨ Patient’s feelings: relieve anxiety and worry about side effects, complications, scars, and disorders after surgery (H) ⑩ Patient’s feelings: empathy and comfort from caregivers, friends, family, and medical staff (H) ⑪⑫ Information delivery method and tool: support short-answer or non-verbal communication methods and tools (F), (M)
(S3-3) Questions and answers	Verbal language (Q&A)	(F) Simple conversational method through short-answer questions	(M) Lack of methods and tools to communicate other than verbally	
(S3-4) Signing consent	Paper form (operation consent)	-	(F) Simple enumerated information system (M) Font size too small to read	⑬ Information delivery method: improve the content system and composition, for effective information delivery (F) ⑭ Information delivery tool: Improved document design so that elderly patients can easily see where to sign (M)
(4)				
(S4-1) Hospitalized for surgery	Verbal language (explanation)	(F) Comprehensive guidance of the treatment process according to the overall flow and sequence	-	① Information content: comprehensive guidance on the overall process of pre-operative treatment (F)
	Patient’s feelings (anxiety and worry)	-	(H) Despair and psychological pain following the diagnosis of relatively rare cancer (H) Concerns about the occurrence of external disorders such as facial depression after surgery	② Patient’s feelings: alleviate feelings of hopelessness, psychological pain, and anxiety (H)
	Treatment cost	(F) Communication and support with medical staff/government organizations (F) Financial support through insurance (F) Government support for medical expenses	(F) High cost of additional treatment and management (F) The cost burden of the inactive elderly	③ Treatment cost: communication and financial support with medical staff/government organizations (F)
(S4-2) Recover after surgery	Verbal language (explanation)	(F) Description of condition that will improve after surgery (F) Provides comprehensive information on the progress after treatment	-	④ Information content: comprehensive information guide on the treatment progress after (F)
	Visual material	(M) Leverage visuals to verify condition mitigation	-	⑤ Information delivery tool: use precise visual data to check the patient’s progress (M)
	Patient’s pain	-	(H) Inconvenience in breathing, conversation, and eating due to pain and difficulty in supplying sufficient nutrition (H) Pain from swelling and pain (H) Lack of interest and countermeasures for the patient’s eating habits and diet	⑥ Patient’s pain: an active response to patient pain after surgery (H) ⑦ A patient’s pain: prepare measures for patient nutrition (H)
	Patient’s feelings (anxiety and worry, relief and will to live)	(H) Positive attitude toward treatment and recovery (H) Depression is relieved when surgery results are good	(H) Concerns about facial deformities after surgery (H) Psychological pain experienced by caregivers during long-term treatment (H) Patient’s burden due to multiple other treatments and repeated evaluation procedure	⑧ Patient’s feelings: support in maintaining a positive attitude toward treatment and recovery (H) ⑨ Patient’s feelings: empathy and support for the psychological pain of the caregiver (H) ⑩ Hospital system: simplify procedures through a flexible link between departments (F)
	Doctor’s attitude	(H) Encouragement and psychological support for patients after surgery	-	⑪ Doctor’s attitude: encouragement and psychological support for patients after surgery (H)
(S4-3) Consulting other departments	Hospital system	(M) Need for information media that can comprehensively guide procedures and contents (M) Communication through text messages between patients, guardians, and medical staff	(F) Lack of connection between medical staff, and the different treatment methods of medical departments (F) Long hospital stay after surgery	⑫ Information delivery tool: information media that can comprehensively guide treatment procedures and contents (M) ⑬ Information delivery tool: a medium for information delivery and communication between patients, guardians, and medical staff (M) ⑭ Hospital system: simplify procedures through a flexible linkage between departments (F)
(5)				
(S5-1) Undergoing rehabilitation	Patient’s pain	-	(H) Difficulty in eating and nutrition for patients after surgery	① Patient’s feelings: encouragement to maintain a hopeful attitude toward treatment (H) ② Caregiver’s feelings: empathy and encouragement for the caregiver’s feelings (H) ③ A patient’s pain: taking care of the patient’s nutritional intake and preparing countermeasures (H)
	Patient’s feelings (depression and burden)	(H) Hopeful attitude toward treatment	(H) The burden of long-term consultation with other departments (H) Difficulty eating properly, causing depression (H) Anxiety about minor symptoms after surgery (H) The burden on caregivers from patient care (5-8a) (H) The burden of self-management of the operative site	
	Verbal language (explanation)	(F) Information on alleviation of postoperative symptoms	(F) Lack of guidance on daily activity after surgery (F) Obtaining conflicting information from other healthcare providers	④ Information content: information related to symptom relief after surgery (F) ⑤ Information content: guidance on daily activity after surgery (F) ⑥ Hospital system: flexible linkage and communication between departments (F)
	Hospital system	(F) Provision of a consultation window for inquiries (F) Provision of administrative assistance for guidance and reservations	(F) Absence of rehabilitation training guidelines	⑦ Hospital system: establish a consultation window for inquiries (F) ⑧ Hospital system: provide administrative assistance for guidance and reservations (F) ⑨ Information content: develop and provide rehabilitation training guidelines (F)
	Education program	(F) Providing education programs on the causes of cancer (F) Providing education programs that anyone can easily access	(F) Lack of programs to keep up with other patient cases or the latest medical technology trends (F) Absence of smoking cessation, sobriety education program	⑩ Education program: provide programs related to the cause of cancer (smoking, abstinence) that anyone can participate in (F) ⑪ Education program: provide a program that can identify cases of cancer overcoming or the latest medical technology trends (F)
(S5-2) Visiting hospital for follow-up	Location /facility	(M) Hospital location close to home, convenient for long-term care (M) Comfortable and pleasant treatment environment	-	⑫ Hospital environment: provide convenient transportation to and from hospitals (M) ⑬ Hospital environment: establishment of a comfortable and pleasant treatment environment (M)
	Website	(M) Providing reliable information over the Internet	(M) Lack of media to obtain additional cancer-related information other than clinical counseling	⑭ Information delivery tool: develop a medium for providing reliable information on cancer (M)
	Patient’s feelings (trust in doctor)	(H) Continuing treatment by building trust (H) Patient’s cooperative attitude after building trust (H) Positive experiences related to previous visits	(H) Passive attitude that makes it challenging to express dissatisfaction	⑮ Patient’s feelings: build trust with doctors (H) ⑯ Patient’s feelings: create an atmosphere in which patients can express their opinions comfortably (H)
	Doctor’s attitude	(H) Doctor’s attitude to explaining calmly (H) Psychological support for patients	-	⑰ Doctor’s attitude: a calm and empathetic attitude of medical staff to relieve anxiety and tension in patients and build trust (H)

(F), functional aspect; (M), mechanic aspect; (H), humanic aspect. (The full version of this Table 3 containing the reference code numbers for each item is provided in Appendix A—Table A1).

**Table 4:** Summary of insights based on the HNC patient needs.

Service Clue	Category	Insights
Functional	Information content	(S1) ** Reliable information delivery on relatively rare cancers (S1) ** Comprehensive guide to the overall treatment process, including the cause of cancer, the necessity and process of the evaluation procedure, postoperative rehabilitation treatment methods, and surgical procedures and duration (S1) Guidance on how to shorten the evaluation period and give options
		(S2) ** Comprehensive guidance on the treatment process, method, and rehabilitation (S2) ** Repeated summary explanation of the surgical method and side effects (S2) Provide clear evidence for treatment methods (S2) Provides guidance and options for treatment methods (S2) Provide successful cancer treatment cases
		(S3) Information delivery considering the hierarchy and system (S3) Advance guidance on treatment direction, postoperative pain management, and intensive care in the ICU (S3) Balanced explanation by topic of information content
		(S4) ** Comprehensive information guide on the treatment progress after surgery (S4) Comprehensive guide to the overall treatment process
		(S5) ** Guide to daily activity after surgery (S5) Information related to symptom relief after surgery (S5) Development and provision of rehabilitation training guidelines
	Information delivery method	(S1–S3) ** Use terms that are easy for the patient to understand
		(S3) An explanation method that points out the operative site (S3) Improvement of content system and composition for effective information delivery
	Hospital system	(S1) ** Establish close linkages between departments to prevent delays
		(S2) Providing support services in consideration of individual patient circumstances (S2) Provision of text message service for the treatment schedule
		(S4) ** Simplification of procedures through the flexible linkage between departments
		(S5) Flexible linkage and communication between departments (S5) Establishment of a counseling window for inquiries (S5) Administrative support for guidance and reservations
	Treatment cost	(S4) Financial support through communication with medical staff/government agencies
	Education program	(S5) ** Provides smoking cessation and alcohol-free programs for public (S5) ** Provides programs to share cancer survivor cases or the latest medical technology
Mechanic	Information delivery tool	(S1) Provision of information delivery media that patients can easily access and use from anywhere
		(S2) Use of visual data that is easy for patients to understand (S2) Provides documentation with a comprehensive description of the treatment process and methods (S2) Support for non-verbal communication tools
		(S3) ** Use of large, clear visuals that are easy to understand and recognizable by older patients (S3) Support for simple and non-verbal communication methods and tools (S3) Improvement of document design so that elderly patients can easily recognize
		(S4) Use of precise visual data to check patient progress (S4) Provision of information media that can comprehensively guide treatment procedures and contents (S4) Provision of media for information delivery and communication between patients, guardians, and medical staff
		(S5) Development of reliable information-providing media related to cancer
	Hospital environment (location, space, and furniture)	(S3) Securing a separate consultation space (S3) Improving furniture arrangement and structure for doctor–patient interactions
		(S5) Providing convenient transportation to and from hospitals (S5) Establishment of a comfortable and pleasant treatment environment
Humanic	Patient’s pain	(S4) Active response to patient’s pain after surgery (S4-S5) Prepare measures for patient nutrition
	Patient’s feelings (including caregivers)	(S1) Alleviating the psychological shock of patients following cancer diagnosis (S1) Relief of anxiety and worry about long-term treatment journey and post-surgery life
		(S2) ** Building trust in doctors (S2) Alleviating patient anxiety about surgery and outcome (S2) Empathy with patient concerns and anxiety (S2) Emotional stability and expression of desire for long-term treatment
		(S3) ** Relief of anxiety and worry about side effects, complications, scars, and disorders after surgery (S3) Empathy and comfort from caregivers, friends, family, and medical staff for patient anxiety, worries, and pain
		(S4) Relief of hopelessness, psychological pain, and anxiety (S4) Support for maintaining a positive attitude toward treatment and recovery (S4) Empathy and support for the psychological pain of the caregiver
		(S5) ** Encouragement of patient to maintain a hopeful attitude toward treatment (S5) Building trust with the doctor (S5) Creating an atmosphere in which patients can express their opinions comfortably (S5) Empathy and encouragement for caregivers’ feelings
	Doctor’s attitude	(S1) ** Inducing patient psychological stability and forming a bond through information sharing and positive attitude toward patients
		(S2) Attitude toward understanding and empathizing with the situation of the patient/caregiver (S2) Communicate objective facts, but ** **maintain a positive attitude**
		(S4) ** Encouragement and psychological support for patients after surgery
		(S5) Calm and empathetic attitude of medical staff to relieve anxiety and tension in patients and build trust

(S), Journey stage, **, Key factors and insight evaluated as relatively necessary for improving HNC PE from the doctor’s feedback. (The full version of this Table 4 containing the reference code numbers for each item is provided in Appendix A—Table A2).

## Data Availability

The data that support the findings of this study are available on request from the corresponding author. The data are not publicly available due to privacy or ethical restrictions.

## Appendix A

**Table A1:** (1) Journey Stage 1: studies for cancer diagnosis and staging. (2) Journey Stage 2: pre-operative counseling. (3) Journey Stage 3: getting operation consent. (4) Journey Stage 4: surgery and recovery. (5) Journey Stage 5: Rehabilitation and follow-up.

Action	Touchpoint	Enablers	Barriers	Insights
(S1-1) Recognizing symptom	Patient’s pain	-	(H) Discomfort in daily life (breathing, eating, talking) according to the location of head and neck cancer (1-7b)	-
	Website	(F) Obtain reliable information from another channel (1-19b) (F) Obtain easy-to-understand information that can be used anytime, anywhere (1-21)	(F) Lack of information about relatively rare cancers (1-5c, 1-20b) (F) Lack of prior information for early detection of cancer (1-10) (M) Lack of information delivery media that allow information to be researched in advance (1-19a) (F) Fear of obtaining misinformation (1-20a)	① Information content: reliable information delivery on relatively rare cancers (1-5c, 1-20b, 1-19b, 1-20a) (F) ② Information delivery tool: provide an information delivery medium that patients can easily access and use anywhere (1-10, 1-19a, 1-21) (M)
(S1-2) Visiting hospital /consulting	Verbal language (explanation)	(F) Advance notice on evaluation procedure (1-12a, 1-13a, 1-13b) (F) Description of further studies for staging (1-12b) (F) Opportunity to choose evaluation procedure (1-12c) (F) Distinguish between evaluation procedure and surgery, and provide clear information on it (1-14b)	(F) Medical term unfamiliar to the patient (1-7a)	③ Information content: guidance and explanation of the need for evaluation procedure (1-12a, 1-12b, 1-13a, 1-13b, 1-14b) (F) ④ Information content: guidance on methods that can shorten the evaluation period (1-12c) (F) ⑤ Information delivery method: use terms that are easy for patients to understand (1-7a) (F)
(S1-3) Running studies for staging	Biopsy, imaging studies	-	(F) A slow-paced evaluation procedure (1-5b, 1-13c, 1-16, 1-17)	⑥ Hospital system: establish close linkage between departments to prevent delays (1-5b, 1-13c, 1-16, 1-17) (F)
(S1-4) Checking the results/being diagnosed with cancer	Verbal language (explanation)	(F) Guidance on method and direction of rehabilitation treatment after surgery (1-8) (F) A guide to the causes of cancer (1-9) (F) Overall surgical method and procedure guide (1-11) (F) Guidance on surgical procedure and duration (1-14c)	(F) Medical term unfamiliar to the patient (1-7a)	⑦ Information content: comprehensive guidance on the cause of cancer, postoperative rehabilitation treatment methods, surgical procedures and duration, and overall treatment process (1-8, 1-9, 1-11, 1-14c) (F) ⑤ Information delivery method: use terms that are easy for patients to understand (1-7a) (F)
	Patient’s feelings (shock and fear)	-	(H) Psychological impact on cancer diagnosis (1-5a) (H) Anxiety and worries about life after surgery (1-6) (H) Concerns about a long treatment journey (1-14a)	⑧ Patient’s feelings: alleviate the psychological shock of patients following cancer diagnosis (1-5a) (H) ⑨ Patient’s feelings: alleviate anxiety and worry about long-term treatment journey and life after surgery (1-6, 1-14a) (H)
	Doctor’s attitude	(H) The optimistic attitude of the medical staff (1-1) (H) Inducing patient stability and psychological support (1-2, 1-4) (H) Building a bond by sharing information about patients (1-15a)	-	⑩ Doctor’s attitude: induce psychological stability and form a bond with the patient by information sharing and positive attitude toward patients (1-1, 1-2, 1-4, 1-15a) (H)
(2)				
(S2-1) Determining treatment methods	Patient’s pain	-	(H) Pain at the site of cancer (2-11)	-
	Evaluation report	(F) Accurate diagnosis with clear evidence (2-50)	-	① Information content: present clear evidence for treatment methods (2-50) (F)
(S2-2) Explaining treatment	Verbal language (explanation)	(F) Clear explanation of surgical methods, results, and rehabilitation (2-3a, 2-14, 2-16a) (F) Comprehensive explanation and general overview of the treatment process and diagnosis (2-13, 2-17b, 2-20) (F) Guidance and selection of treatment methods (2-33) (F) Use easy-to-understand terminology (2-35b, 2-51a, 2-51b)	(F) Lack of understanding of older patients for unfamiliar and complex content (2-16b, 2-60b) (F) Different level of understanding by caregiver (2-60a)	② Information content: a comprehensive explanation of the treatment process, method, and rehabilitation (2-3a, 2-13, 2-14, 2-16a, 2-17b, 2-20) (F) ③ Information content: provide guidance and options for bd treatment methods (2-33) (F) ④ Information delivery method: use terminology that is easy for the patient/caregiver to understand (2-35b, 2-51a, 2-51b) (F)
	Body language	(M) Description of surgical methods using body language (2-17a, 2-34, 2-36)	-	⑤ Information delivery tool: use visual data that is easy for patients to understand (2-17a, 2-34, 2-35a, 2-35b, 2-36, 2-38b, 2-42)/provide documentation with a comprehensive description of the treatment process and methods (2-17b) (M)
	Visual material	(M) Check the location of the cancer with an endoscope (2-38a) (M) Description of operative site and surgical plan using image data (2-41) (M) Provide easy-to-understand visuals (2-35b)	(M) Need to provide comprehensive documentation (2-17b) (M) Image data that are difficult for the patient to understand (2-35a, 2-38b, 2-42)	
(S2-3) Discussing treatment	Verbal Language (Q & A)	(F) A guide to the need for caregivers (2-4a, 2-21a) (F) Services that identify and support the patient’s situation and concerns (2-4b, 2-8b, 2-21c, 2-24b) (F) Access to successful cancer treatment practices (2-10a, 2-10b) (F) Sharing sufficient information with both patients and caregivers (2-12)	(F) Lack of way to discuss caregivers in advance (2-24a) (F) Inconvenient handwriting due to language communication difficulties (2-37a, 2-40) (M) Lack of communication tools (2-37b)	⑥ Hospital system: provide support services that consider the individual circumstances of the patient (2-4a, 2-4b, 2-8b, 2-21a, 2-21c, 2-24a, 2-24b) (F) ⑦ Information content: provide successful cancer treatment cases (2-10a, 2-10b) (F) ⑧ Information delivery tool: support non-language communication tools (2-37a, 2-37b, 2-40) (M)
	Patient’s Feelings (anxiety and worry)	-	(H) Anxiety about the patient’s surgical procedure and results (2-2b, 2-3a, 2-5a, 2-5b, 2-7a, 2-57c) (H) Lack of empathy for patient care or concern and anxiety (2-4c, 2-6, 2-24c, 2-63) (H) Anxiety and worries from long-term treatment (2-7b, 2-45, 2-57b) (H) Patient’s impatience for quick treatment (2-58b)	⑨ Patient’s feelings: alleviate patient anxiety about surgery and outcomes (2-2b, 2-3a, 2-5a, 2-5b, 2-7a, 2-7b, 2-45, 2-57b, 2-57c, 2-58b) (H) ⑩ Patient’s feelings: empathy for the patient’s concerns and anxiety (2-4c, 2-6, 2-24c, 2-63) (H)
	Doctor’s attitude	(H) Attitude to understand and empathize with the situation of a patient without a caregiver (2-21b) (H) Empathize with the situation and psychological aspects of the patient or caregiver (2-54) (H) Kind and empathetic explanations and attitudes (2-8a, 2-56)	(H) Need for positive attitudes and words to be provided with objective facts (2-44, 2-46, 2-47a, 2-48a, 2-48c, 2-48d)	⑪ Doctor’s attitude: understand and empathize with the situation of the patient/caregiver (2-8a, 2-21b, 2-54, 2-56) (H) ⑫ Doctor’s attitude: positive attitude and words when conveying objective facts (2-44, 2-46, 2-47a, 2-48a, 2-48c, 2-48d) (H)
(S2-4) Deciding and planning the schedule	Verbal language (explanation)	(F) Summary of surgery methods and side effects (2-18, 2-49a) (F) Sharing other patient cases (2-53b)	-	⑬ Information content: repeated summary explanations of surgical methods and side effects (2-18, 2-49a) (F) ⑭ Information content: share cases with other patients (2-53b) (F)
	Hospital system	(F) Text message service for hospital schedules (2-61a, 2-61b)	(F) Long-awaited surgery schedule (2-58a)	⑮ Hospital system: provide a text message service for the treatment schedule (2-61a, 2-61b) (F)
	Patient’s feelings (trust)	(H) Building trust in doctors’ experience (2-52, 2-55) (H) Importance of emotional stability and will for long-term treatment (2-53a) (H) Satisfaction and reliability through easy understanding (2-51c)	-	⑯ Patient’s feelings: build trust in doctors (2-51c, 2-52, 2-55) (H) ⑰ Patient’s feelings: emotional stability and expression of will for long-term treatment (2-53a) (H)
(3)				
(S3-1) Entering room	Consult room	-	(M) Environment where it is difficult to concentrate on explanation due to ambient noise (steps, conversation) (3-1b) (M) Arrangement of furniture that makes doctor–patient interaction difficult (3-32b, 3-39b, 3-40b, 3-41b)	① Space and furniture arrangement: a separate medical space (3-1b) (M) ② Space and furniture arrangement: improve furniture arrangement so that doctors-patients can face each other and focus on paperwork (3-32b, 3-39b, 3-40b, 3-41b) (M)
(S3-2) Explaining surgery process and method	Verbal language (explanation)	(F) Description of treatment direction (3-28e) (F) Explain medical terms in an easy-to-understand manner (3-13e, 3-24e) (F) Realistic level of advice (3-23e) (F) Describe how to manage pain after surgery (3-7e) (F) Information on intensive care in the ICU after surgery (3-10e)	(F) A large amount of information that is difficult to hear and remember at once (3-16b) (F) Explanation of the process too detailed and specific (3-6b) (F) Lack of detailed management guidance (3-14b) (F) Comprehensive and lengthy description of complications (3-23b)	③ Information content: information delivery considering the hierarchy and system of information content (3-6b, 3-14b, 3-16b) (F) ④ Information content: prior guidance on treatment direction, postoperative pain management, and intensive care in the ICU (3-28e, 3-7e, 3-10e, 3-23e) (F) ⑤ Information content: a balanced explanation by topic of information content (3-6b, 3-14b, 3-23b) (F) ⑥ Information delivery method: use easy-to-understand terminology (3-13e, 3-24e) (F)
	Body language	(F) Direct explanation through re-enactment while pointing to body parts (3-15e)	-	⑦ Information delivery method: explanation clearly pointing out the operative site (3-15e) (F)
	Hand drawings	-	(M) Hard-to-understand hand-drawn explanations (3-2b) (M) Hand drawing difficult to convey exact meaning (3-30b, 3-32b) (M) Small hand drawings that are difficult for elderly patients to see (3-31b)	⑧ Information delivery tool: use large, clear visuals that are easy to understand and recognizable by older patients (3-2b, 3-30b, 3-31b, 3-32b, 3-37b, 3-41b) (M)
	Image data	-	(M) Hard-to-recognize specialized material (CT) (3-37b)	
	Patient’s feelings (anxiety and worry)	-	(H) Difficulty and frustration in communication due to inability to communicate verbally (3-1b) (H) Patient’s psychological burden for surgery (3-2b, 3-3b, 3-5b) (H) Patient’s psychological pain and burden due to metastasis (3-4b) (H) Worry and anxiety about not knowing the treatment plan after surgery in advance (3-7b) (H) Anxiety due to complications (3-8b) (H) Depression about scars or disability that will remain after surgery (3-11b, 3-42b) (H) Lack of emotional support (3-6b)	⑨ Patient’s feelings: relieve anxiety and worry about side effects, complications, scars, and disorders after surgery (3-2b, 3-3b, 3-5b, 3-8b, 3-11b, 3-42b) (H) ⑩ Patient’s feelings: empathy and comfort from caregivers, friends, family, and medical staff (3-4b, 3-6b, 3-7b) (H) ⑪⑫ Information delivery method and tool: support short-answer or non-verbal communication methods and tools (3-22b, 3-29b) (F), (M)
(S3-3) Questions and answers	Verbal language (Q&A)	(F) Simple conversational method through short-answer questions (3-22e, 3-29e)	(M) Lack of methods and tools to communicate other than verbally (3-22b, 3-29b)	
(S3-4) Signing consent	Paper form (operation consent)	-	(F) Simple enumerated information system (3-17b) (M) Font size too small to read (3-41a)	⑬ Information delivery method: improve the content system and composition for effective information delivery (3-16b, 3-17b) (F) ⑭ Information delivery tool: Improved document design so that elderly patients can easily see where to sign (3-41a) (M)
(4)				
(S4-1) Hospitalized for surgery	Verbal language (explanation)	(F) Comprehensive guidance of the treatment process according to the overall flow and sequence (4-12)	-	① Information content: comprehensive guidance on the overall process of pre-operative treatment (4-12) (F)
	Patient’s feelings (anxiety and worry)	-	(H) Despair and psychological pain following the diagnosis of a relatively rare cancer (4-4) (H) Concerns about the occurrence of external disorders such as facial depression after surgery (4-27b, 4-35)	② Patient’s feelings: alleviate feelings of hopelessness, psychological pain, and anxiety (4-4, 4-27b, 4-35) (H)
	Treatment cost	(F) Communication and support with medical staff/government organizations (4-11) (F) Financial support through insurance (4-27a) (F) Government support for medical expenses (4-28a)	(F) High cost of additional treatment and management (4-26a, 4-26b, 4-27b, 4-29b) (F) The cost burden of the inactive elderly (4-28b)	③ Treatment cost: communication and financial support with medical staff/government organizations (4-11, 4-27a, 4-28a, 4-26a, 4-26b, 4-27b, 4-29b, 4-28b) (F)
(S4-2) Recover after surgery	Verbal language (explanation)	(F) Description of condition that will improve after surgery (4-1, 4-14a, 4-14b) (F) Provides comprehensive information on the progress after treatment (4-14c, 4-31a)	-	④ Information content: comprehensive information guidance on the treatment progress after surgery (4-1, 4-14a, 4-14b, 4-14c, 4-31a) (F)
	Visual material	(M) Leverage visuals to verify condition mitigation (4-17, 4-18)	-	⑤ Information delivery tool: use clear visual data to check the patient’s progress (4-17, 4-18) (M)
	Patient’s pain	-	(H) Inconvenience in breathing, conversation, and eating due to pain and difficulty in supplying sufficient nutrition (4-5, 4-7a, 4-25b) (H) Pain from swelling and pain (4-7b) (H) Lack of interest and countermeasures for the patient’s eating habits and diet (4-25a)	⑥ Patient’s pain: an active response to patient pain after surgery (4-7b) (H) ⑦ A patient’s pain: prepare measures for patient nutrition (4-5, 4-7a, 4-25a, 4-25b) (H)
	Patient’s feelings (anxiety and worry, relief and will to live)	(H) Positive attitude toward treatment and recovery (4-2b) (H) Depression is relieved when surgery results are good (4-8, 4-33, 4-34)	(H) Concerns about facial deformities after surgery (4-2a) (H) Psychological pain experienced by caregivers during long-term treatment (4-9, 4-10) (H) Patient’s burden due to multiple other treatments and repeated evaluation procedure (4-29a, 4-30b)	⑧ Patient’s feelings: support in maintaining a positive attitude toward treatment and recovery (4-2b) (H) ⑨ Patient’s feelings: empathy and support for the psychological pain of the caregiver (4-9, 4-10) (H) ⑩ Hospital system: simplify procedures through flexible link between departments (4-29a, 4-30b) (F)
	Doctor’s attitude	(H) Encouragement and psychological support for patients after surgery (4-6)	-	⑪ Doctor’s attitude: encouragement and psychological support for patients after surgery (4-6) (H)
(S4-3) Consulting other departments	Hospital system	(M) Need for information media that can comprehensively guide procedures and contents (4-13) (M) Communication through text messages between patients, guardians, and medical staff (4-22)	(F) Lack of connection between medical staff and different treatment methods of medical departments (4-30a, 4-30c, 4-31b) (F) Long hospital stay after surgery (4-19, 4-20)	⑫ Information delivery tool: information media that can comprehensively guide treatment procedures and contents (4-13) (M) ⑬ Information delivery tool: a medium for information delivery and communication between patients, guardians, and medical staff (4-22) (M) ⑭ Hospital system: simplify procedures through flexible linkage between departments (4-19, 4-20, 4-30a, 4-30c, 4-31b) (F)
(5)				
(S5-1) Undergoing rehabilitation	Patient’s pain	-	(H) Difficulty in eating and nutrition in patients after surgery (5-1, 5-6b)	① Patient’s feelings: encouragement patients to maintain a hopeful attitude toward treatment (5-3a, 5-4, 5-7, 5-8b) (H) ② Caregiver’s feelings: empathy and encouragement for the caregiver’s feelings (5-8a) (H) ③ A patient’s pain: taking care of the patient’s nutritional intake and preparing countermeasures (5-1, 5-3b, 5-6b, 5-6c) (H)
	Patient’s feelings (depression and burden)	(H) Hopeful attitude toward treatment (5-3a)	(H) The burden of long-term consultation of other department (5-6a) (H) Difficulty eating properly, causing depression (5-3b, 5-6c) (H) Anxiety about minor symptoms after surgery (5-4) (H) The burden on caregivers for patient care (5-8a) (H) The burden of self-management of the operative site (5-7, 5-8b)	
	Verbal language (explanation)	(F) Information on alleviation of postoperative symptoms (5-11)	(F) Lack of guidance on daily activity after surgery (5-22, 5-24) (F) Obtaining conflicting information from other health care providers (5-25)	④ Information content: information related to symptom relief after surgery (5-11) (F) ⑤ Information content: guidance on daily activity after surgery (5-22, 5-24) (F) ⑥ Hospital system: flexible linkage and communication between departments (5-25) (F)
	Hospital system	(F) Provision of a consultation window for inquiries (5-10) (F) Provision of administrative assistance for guidance and reservations (5-20)	(F) Absence of rehabilitation training guidelines (5-21)	⑦ Hospital system: establish a consultation window for inquiries (5-10) (F) ⑧ Hospital system: provide administrative assistance for guidance and reservations (5-20) (F) ⑨ Information content: develop and provide rehabilitation training guidelines (5-21) (F)
	Education program	(F) Providing education programs on the causes of cancer (5-23a) (F) Providing education programs that anyone can easily access (5-23c)	(F) Lack of programs to keep up with other patient cases or the latest medical technology trends (5-19) (F) Absence of smoking cessation, sobriety education program (5-23b)	⑩ Education program: provide programs related to the cause of cancer (smoking, abstinence) that anyone can participate in (5-23a, 5-23b, 5-23c) (F) ⑪ Education program: provide a program that can identify cases of cancer overcoming or the latest medical technology trends (5-19) (F)
(S5-2) Visiting hospital for follow-up	Location /facility	(M) Hospital location close to home, convenient for long-term care (5-16) (M) Comfortable and pleasant treatment environment (5-18)	-	⑫ Hospital environment: provide convenient transportation to and from hospitals (5-16) (M) ⑬ Hospital environment: establishment of a comfortable and pleasant treatment environment (5-18) (M)
	Website	(M) Providing reliable information over the Internet (5-17b)	(M) Lack of media to obtain additional cancer-related information other than clinical counseling (5-17a)	⑭ Information delivery tool: develop a medium for providing reliable information on cancer (5-17a, 5-17b) (M)
	Patient’s feelings (trust in doctor)	(H) Continuing treatment by building trust (5-12b) (H) Patient’s cooperative attitude after building trust (5-13) (H) Positive experiences related to previous visits (5-14, 5-15)	(H) Passive attitude that makes it difficult to express dissatisfaction (5-2)	⑮ Patient’s feelings: build trust with doctors (5-12b, 5-13, 5-14, 5-15) (H) ⑯ Patient’s feelings: create an atmosphere in which patients can express their opinions comfortably (5-2) (H)
	Doctor’s attitude	(H) Doctor’s attitude to explain calmly (5-12a) (H) Psychological support for patients (5-3c)	-	⑰ Doctor’s attitude: a calm and empathetic attitude of medical staff to relieve anxiety and tension in patients and build trust (5-3c, 5-12a) (H)

(F), functional aspect; (M), mechanic aspect; (H), humanic aspect.

**Table A2:** Summary of insights based on the HNC patient needs.

Service Clue	Category	Insights
Functional	Information content	**(S1) ** Reliable information delivery on relatively rare cancers** (1-5c, 1-20b, 1-19b, 1-20a) **(S1) ** Comprehensive guide to the overall treatment process**, including the cause of cancer, the necessity and process of evaluation procedure, postoperative rehabilitation treatment methods, and surgical procedures and duration (1-8, 1-9, 1-11, 1-12a, 1-12b, 1-13a, 1-13b, 1-14b, 1-14c) (S1) Guidance on how to shorten the evaluation period and giving options (1-12c)
		**(S2) ** Comprehensive guidance on treatment process, method, and rehabilitation** (2-3a, 2-13, 2-14, 2-16a, 2-17b, 2-20) **(S2) ** Repeated summary explanation** of the surgical method and side effects (2-18, 2-49a) (S2) Provide clear evidence for treatment methods (2-50) (S2) Provides guidance and options for treatment methods (2-33) (S2) Provide successful cancer treatment cases (2-10a, 2-10b, 2-53b)
		(S3) Information delivery considering the hierarchy and system (3-6b, 3-14b, 3-16b) (S3) Advance guidance on treatment direction, postoperative pain management, and intensive care in the ICU (3-28e, 3-7e, 3-10e, 3-23e) (S3) Balanced explanation by topic of information content (3-6b, 3-14b, 3-23b)
		**(S4) ** Comprehensive information guide on the treatment progress after surgery** (4-1, 4-14a, 4-14b, 4-14c, 4-31a) (S4) Comprehensive guide to the overall treatment process (4-12)
		**(S5) ** Guide to daily activity after surgery** (5-22, 5-24) (S5) Information related to symptom relief after surgery (5-11) (S5) Development and provision of rehabilitation training guidelines (5-21)
	Information delivery method	**(S1) ** Use terms that are easy for the patient to understand** (1-7a)
		**(S2) ** Use terms that are easy for the patient to understand** (2-35b, 2-51a, 2-51b)
		**(S3) ** Use terms that are easy for the patient to understand** (3-13e, 3-24e) (S3) An explanation method that clearly points out the operative site (3-15e) (S3) Improvement of content system and composition for effective information delivery (3-16b, 3-17b)
	Hospital system	**(S1) ** Establish close linkage between departments** to prevent delays (1-5b, 1-13c, 1-16, 1-17)
		(S2) Providing support services in consideration of individual patient circumstances (2-4a, 2-4b, 2-8b, 2-21a, 2-21c, 2-24a, 2-24b) (S2) Provision of text message service for treatment schedule (2-61a, 2-61b)
		**(S4) ** Simplification of procedures through flexible linkage between departments** (4-19, 4-20, 4-30a, 4-30c, 4-31b)
		(S5) Flexible linkage and communication between departments (5-25) (S5) Establishment of a counseling window for inquiries (5-10) (S5) Administrative support for guidance and reservations (5-20)
	Treatment cost	(S4) Financial support through communication with medical staff/government agencies (4-11, 4-27a, 4-28a, 4-26a, 4-26b, 4-27b, 4-29b, 4-28b)
	Education program	**(S5) ** Provides smoking cessation and alcohol-free programs** that anyone can participate in (5-23a, 5-23b, 5-23c) **(S5) ** Provides programs to share cancer overcoming cases or the latest medical technology trends** (5-19)
Mechanic	Information delivery tool	(S1) Provision of information delivery media that patients can easily access and use from anywhere (1-10, 1-19a, 1-21)
		(S2) Use of visual data that is easy for patients to understand (2-17a, 2-34, 2-35a, 2-35b, 2-36, 2-38b, 2-42) (S2) Provides documentation with a comprehensive description of the treatment process and methods (2-17b) (S2) Support for non-verbal communication tools (2-37a, 2-37b, 2-40)
		**(S3) ** Use of large, clear visuals** that are easy to understand and recognizable by older patients (3-2b, 3-30b, 3-31b, 3-32b, 3-37b, 3-41b) (S3) Support for simple and non-verbal communication methods and tools (3-22b, 3-29b) (S3) Improvement of document design that can be easily recognized by elderly patients (3-41a)
		(S4) Use of clear visual data to check patient progress (4-17, 4-18) (S4) Provision of information media that can comprehensively guide treatment procedures and contents (4-13) (S4) Provision of media for information delivery and communication between patients, guardians, and medical staff (4-22)
		(S5) Development of reliable information-providing media related to cancer (5-17a, 5-17b)
	Hospital environment (location, space, and furniture)	(S3) Securing a separate consultation space (3-1b) (S3) Improving furniture arrangement and structure for doctor–patient interaction (3-32b, 3-39b, 3-40b, 3-41b)
		(S5) Providing convenient transportation to and from hospitals (5-16) (S5) Establishment of a comfortable and pleasant treatment environment (5-18)
Humanic	Patient’s pain	(S4) Active response to patient’s pain after surgery (4-7b) (S4) Prepare measures for patient nutrition (4-5, 4-7a, 4-25a, 4-25b)
		(S5) Prepare measures for patient nutrition (5-1, 5-3b, 5-6b, 5-6c)
	Patient’s feelings (including caregivers)	(S1) Alleviating the psychological shock of patients following cancer diagnosis (1-5a) (S1) Relief of anxiety and worry about long-term treatment journey and post-surgery life (1-6, 1-14a)
		**(S2) ** Building trust in doctors** (2-51c, 2-52, 2-55) (S2) Alleviating patient anxiety about surgery and outcome (2-2b, 2-3a, 2-5a, 2-5b, 2-7a, 2-7b, 2-45, 2-57b, 2-57c, 2-58b) (S2) Empathy with patient concerns and anxiety (2-4c, 2-6, 2-24c, 2-63) (S2) Emotional stability and expression of will for long-term treatment (2-53a)
		**(S3) ** Relief of anxiety and worry about side effects, complications, scars, and disorders after surgery** (3-2b, 3-3b, 3-5b, 3-8b, 3-11b, 3-42b) (S3) Empathy and comfort from caregivers, friends, family, and medical staff for patient anxiety, worries, and pain (3-4b, 3-6b, 3-7b)
		(S4) Relief of hopelessness, psychological pain, and anxiety (4-4, 4-27b, 4-35) (S4) Support for maintaining a positive attitude toward treatment and recovery (4-2b) (S4) Empathy and support for the psychological pain of the caregiver (4-9, 4-10)
		**(S5) ** Encouragement to maintain a patient’s hopeful attitude toward treatment** (5-3a, 5-4, 5-7, 5-8b) (S5) Building trust with doctor (5-12b, 5-13, 5-14, 5-15) (S5) Creating an atmosphere in which patients can express their opinions comfortably (5-2) (S5) Empathy and encouragement for caregivers’ feelings (5-8a)
	Doctor’s attitude	**(S1) ** Inducing patient psychological stability and forming a bond through information sharing and positive attitude toward patients** (1-1, 1-2, 1-4, 1-15a)
		(S2) Attitude to understand and empathize with the situation of the patient/caregiver (2-8a, 2-21b, 2-54, 2-56) (S2) Communicate objective facts, but ****maintain a positive attitude** (2-44, 2-46, 2-47a, 2-48a, 2-48c, 2-48d)
		**(S4) ** Encouragement and psychological support for patients after surgery** (4-6)
		(S5) Calm and empathetic attitude of medical staff to relieve anxiety and tension in patients and build trust (5-3c, 5-12a)

(S), Journey stage, **, Key factors and insight evaluated as relatively important for improving HNC PE from the doctor’s feedback.
